# Functional exploration of co-expression networks identifies a nexus for modulating protein and citric acid titres in *Aspergillus niger* submerged culture

**DOI:** 10.1186/s40694-019-0081-x

**Published:** 2019-11-09

**Authors:** Timothy C. Cairns, Claudia Feurstein, Xiaomei Zheng, Li Hui Zhang, Ping Zheng, Jibin Sun, Vera Meyer

**Affiliations:** 1 Tianjin Institute of Industrial Biotechnology, Chinese Academy of Sciences, Tianjin, 300308 People’s Republic of China; 2 Key Laboratory of Systems Microbial Biotechnology, Chinese Academy of Sciences, Tianjin, 300308 People’s Republic of China; 3 Institute of Biotechnology, Chair of Applied and Molecular Microbiology, Technische Universität Berlin, Straße des 17. Juni 135, 10623 Berlin, Germany; 4 University of Chinese Academy of Sciences, Beijing, 100049 China; 5 College of Biotechnology, Tianjin University of Science & Technology, Tianjin, 300457 China

**Keywords:** Protein secretion, Citric acid, *Aspergillus niger*, Pellet, CRISPR, Dispersed mycelia, Polar growth, Arf, Tet-on, Genome editing

## Abstract

**Background:**

Filamentous fungal cell factories are used to produce numerous proteins, enzymes, and organic acids. Protein secretion and filamentous growth are tightly coupled at the hyphal tip. Additionally, both these processes require ATP and amino acid precursors derived from the citric acid cycle. Despite this interconnection of organic acid production and protein secretion/filamentous growth, few studies in fungi have identified genes which may concomitantly impact all three processes.

**Results:**

We applied a novel screen of a global co-expression network in the cell factory *Aspergillus niger* to identify candidate genes which may concomitantly impact macromorphology, and protein/organic acid fermentation. This identified genes predicted to encode the Golgi localized ArfA GTPase activating protein (GAP, AgeB), and ArfA guanine nucleotide exchange factors (GEFs SecG and GeaB) to be co-expressed with citric acid cycle genes. Consequently, we used CRISPR-based genome editing to place the titratable Tet-on expression system upstream of *ageB*, *secG*, and *geaB* in *A. niger*. Functional analysis revealed that *ageB* and *geaB* are essential whereas *secG* was dispensable for early filamentous growth. Next, gene expression was titrated during submerged cultivations under conditions for either protein or organic acid production. ArfA regulators played varied and culture-dependent roles on pellet formation. Notably, *ageB* or *geaB* expression levels had major impacts on protein secretion, whereas *secG* was dispensable. In contrast, reduced expression of each predicted ArfA regulator resulted in an absence of citric acid in growth media. Finally, titrated expression of either GEFs resulted in an increase in oxaloacetic acid concentrations in supernatants.

**Conclusion:**

Our data suggest that the Golgi may play an underappreciated role in modulating organic acid titres during industrial applications, and that this is SecG, GeaB and AgeB dependent in *A. niger*. These data may lead to novel avenues for strain optimization in filamentous fungi for improved protein and organic acid titres.

## Background

Filamentous fungi are used in diverse biotechnological applications for the production of organic acids, secondary metabolites, enzymes, and proteins [1–3]. Currently, the majority of industrial strains with optimized performance have been generated by mutagenesis screens, resulting in elevated product titres, the utilization of a greater variety of cheap nutrient sources, development of optimized morphologies for improved rheological performance in submerged fermentation, or enhanced resistance to toxic metabolic intermediates, amongst many other desired phenotypes [2, 4]. However, a significant limitation to mutagenesis approaches is that the molecular basis of strain optimisation is extremely difficult to reverse engineer [5], and thus favourable attributes of production strains cannot be easily applied to different isolates or fungal species [4].

Advances in fungal genomic, transcriptomic, and metabolomic datasets have enabled a drastic improvement in the predictive capabilities of fungal biotechnologists, both at the level of individual gene or protein components, but also at the level of integrated systems [6–9]. Recently, we have demonstrated that co-expression networks in the organic acid, secondary metabolite, and protein production factory *A. niger* can be used for novel biotechnological leads [10]. Specifically, we conducted a meta-analysis of over 283 publicly available micro-array datasets, covering 155 different cultivation conditions of *A. niger*, after which co-expression networks were generated at an individual gene level [10]. These co-expression networks can be used to generate novel hypotheses regarding gene function, based on the so called ‘guilt by association’ hypothesis, whereby genes with robust co-expression profiles over sufficiently diverse conditions can be hypothesised to be involved in similar, or the same, biological processes or pathways [11, 12]. Using this approach in our previous study, two so far unknown globally acting transcription factors MjkA and MjkB were identified, which likely control numerous natural product biosynthetic gene clusters in *A. niger* [10].

In the current study, we hypothesised that further exploration of *A. niger* co-expression networks could also be used to identify gene(s) which are highly conserved amongst filamentous fungi that can be used to concomitantly modulate secretion and/or production of the two other classes of industrially relevant products, specifically secreted proteins and organic acids. Indeed, recent fungal metabolomic experiments have predicted that organic acid, protein, and natural product synthesis share numerous fundamental metabolic pathways, biological processes, and sub-cellular components which could be re-engineered during strain optimisation efforts [13]. For example, the tricarboxylic acid (TCA) cycle involves the formation of citric acid from oxaloacetate, acetyl-CoA, and water by a citrate synthase, which ultimately generates chemical energy in the form of adenosine triphosphate (ATP) following oxidative phosphorylation. Obviously, the TCA cycle is a prerequisite for industrial fermentation of organic acids, including citric acid. Additionally, protein secretion via vesicle trafficking along microtubules and actin cables to the hyphal apex is high in ATP demand [14–17]. Moreover, TCA cycle intermediates are used as precursors for amino acid biosynthesis. Possible molecular links between these processes have not been explored from a biotechnological perspective. Consequently, studies which identify candidate targets for optimisation of multiple product classes are currently lacking for fungal cell factories.

**Fig. 1:**
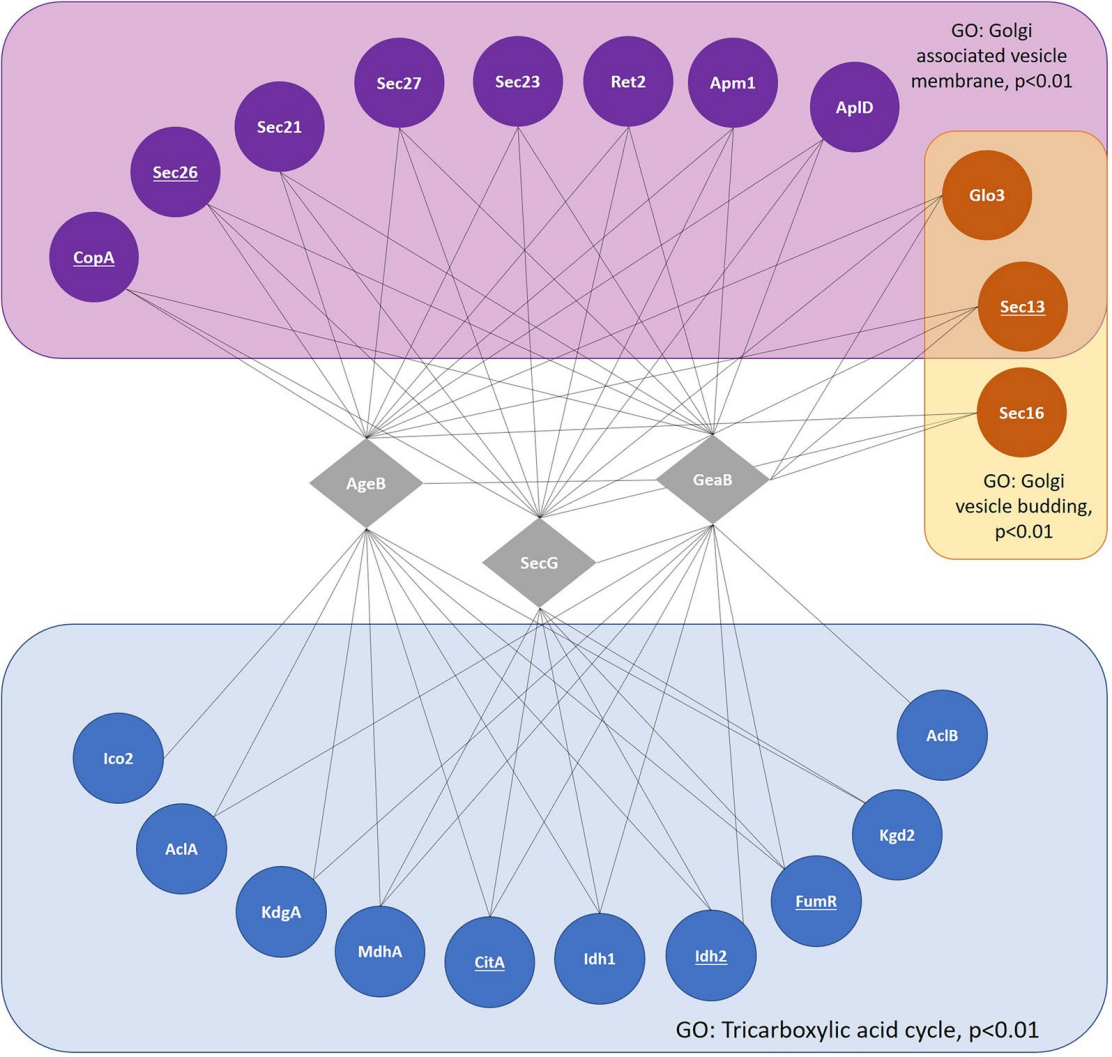
Co-expression networks for a*geB*, s*ecG*, and *geaB* reveal transcriptional coupling of these genes with Golgi vesicle trafficking and tricarboxylic acid cycle in *A. niger*. Query genes are given in grey diamonds, with co-expressed genes depicted as coloured circles. Genes used in multi-gene co-expression analysis are underlined. Grey lines indicate co-expression values above a Spearman cut-off of 0.5, which gives high confidence of robust co-expression throughout > 250 microarray experiments [10]. Examples of enriched GO-terms common to a*geB*, s*ecG*, and *geaB* co-expression networks relative to the *A. niger* genome are depicted. Gene name nomenclature from *A. niger* or the reference organism *A. nidulans* is given, and where not available, from *S. cerevisiae.* Benjamini–Hochberg false discovery rate corrected p-values for GO-enrichment are reported. As expected, these data show clear association of these genes with vesicles at the Golgi and with the TCA cycle

We thus applied a novel in silico approach in which we interrogated the genome-wide co-expression network for *A. niger* [10] for genes encoding proteins which act either in the TCA cycle or at the Golgi. This latter organelle was chosen due to the well documented role of the Golgi in controlling protein secretion and polar growth in filamentous fungi, which may thus also offer avenues for optimizing fungal macromorphology for more efficient fermentation [1]. This in silico interrogation of the genome-wide co-expression resource indeed identified that both cellular functions are transcriptionally coupled with the open reading frames An07g02190, An07g02190 and An11g02650 (Fig. 1). These are orthologs of S*accharomyces cerevisiae* genes *SEC7* (An07g02190), *GEA2* (An18g02490), and *AGE2* (An11g02650), respectively, that encode regulators of the small ADP ribosylation (Arf) GTPases Arf1/2.

Arf GTPases are central to the function of the Golgi, where they regulate a diverse range of processes that have been well described in *S. cerevisiae* (Arf1/2) and *A. niger* (ArfA), including vesicle formation and trafficking, cytoskeletal rearrangements, cell polarity, and morphology [18–21]. Arf1/2 play a critical role in the formation of vesicle coats at distinct steps in intracellular vesicle trafficking in the Golgi, specifically formation of COPI vesicles and clathrin coated vesicles at *cis* and *trans* Golgi cisternae in *S. cerevisiae* [18, 20]. We have previously demonstrated that *A. niger arfA* complements the *ARF1/2* lethal phenotype in *S. cerevisiae*, hence it is a functional equivalent of Arf1/2 [21]. We have furthermore shown that ArfA is important for proper actin ring localisation at hyphal tips of *A. niger* and thus impacts hyphal growth rate, hyphal tip morphology and protein secretion [21]. ArfA cycles between an active GTP-bound and inactive GDP-bound states due to regulation by guanine nucleotide exchange factors (GEFs, Sec7, Gea2) and GTPase activating proteins (GAPs, Age2) [21–23]. Based on our co-expression analysis, we speculated that these ArfA regulators may functionally couple protein and citric acid titres during fermentation, and named ORFs An07g02190, An18g02490 and An11g02650 *secG*, *geaB*, and *ageB*, respectively.

In order to functionally assess if these putative ArfA regulator proteins could indeed be used to modulate protein and acid titres, we used genome editing in *A. niger* [24] to place the Tet-on inducible promoter system [25] upstream of *ageB, secG* or *geaB*. Phenotypic screening of conditional expression mutants on solid media demonstrated that *ageB* and *geaB* are dispensable for germination but essential for hyphal branching and thus colony growth. In contrast, *secG* mutants produced hyphae with length and branch rates which were broadly comparable to the progenitor strain. Consistent with the role of all predicted ArfA GAP and GEFs in vesicle trafficking, lowered expression of each gene resulted in sensitivity to abiotic perturbation which generate unfolded protein stress. Next, we modulated gene expression during shake flask cultures, and quantified morphology and protein/organic acid concentrations, which demonstrated that titres of both classes of metabolites could be modified by *ageB*, *secG* and *geaB* expression levels. These data suggest that genes encoding these predicted ArfA regulatory proteins can indeed be used to modulate *A. niger* morphology and total protein or acid titres during submerged growth.

## Results

### Multi-gene co-expression network analysis suggests the TCA cycle and protein secretion are transcriptionally coupled with *ageB*, *secG*, and *geaB*

We reasoned that *A. niger* gene co-expression networks, which we made publicly available at the data repository FungiDB [6, 10], could be mined for genes which are transcriptionally coupled with protein secretion and citric acid production. Accordingly, we retrieved candidates which are co-expressed with genes involved in the TCA cycle (citrate synthase *citA*, fumarate reductase *fumR*, and isocitrate dehydrogenase *idh2*), and vesicle trafficking at the Golgi (alpha/beta subunits of the coat protein complex (COPI) *copA*/*sec26*, and COPII subunit *sec13*). The COPI and COPII complex mediate retrograde and anterograde vesicle trafficking between the Golgi and endoplasmic reticulum, respectively [26, 27]. Note that all six query genes were also selected due to evidence of function based on wet-lab experimentation conducted in either *A. niger* or *A. nidulans* [28]. Interrogation of co-expression networks above the stringent 0.5 Spearman correlation coefficient threshold revealed that 259 genes were co-expressed with all six query genes. GO enrichment of this multi-gene sub-network suggested the TCA cycle and Golgi vesicle transport are transcriptionally coupled with various processes in *A. niger*, including oxoacid acid/carboxylic acid metabolism, microtubule cytoskeleton organization, hyphal growth, and responses to pH, amongst others (Additional file 1). A notable observation from the GO analysis was enrichment of genes for regulation of Arf protein signal transduction (p = 0.01) which included orthologues for *S. cerevisiae* Arf GEFs SEC7 (An07g02190) and GEA2 (An18g02490, Additional file 1). Manual interrogation of the sub-network also revealed a gene predicted to encode the orthologue for *S. cerevisiae* Arf GTPase activating protein Age2 (An11g02650) co-expressed with all 6 query genes (according to *A. niger* nomenclature, we name these genes *secG*, *geaB* and *ageB* in *A. niger,* respectively). Based on the co-expression network, we hypothesised *A. niger ageB*, *secG*, and *geaB* genes may concomitantly impact protein secretion and organic acid synthesis in *A. niger*.

In order to provide further evidence to support this hypothesis, we conducted a second analysis of co-expression resources, where we interrogated individual *ageB*, *secG*, and *geaB* co-expression networks (Fig. 1 and Table 1). As expected, these sub-networks were highly enriched for GO processes associated with vesicle trafficking at the Golgi including, but not limited to Golgi associated vesicle membrane (GO:0030660, p < 0.01) and Golgi vesicle budding (GO:0048194, p < 0.01, Fig. 1, Additional file 2). Additionally, GO enrichment analysis for *ageB*, *secG*, and *geaB* networks revealed robust co-expression of numerous genes associated with the tricarboxylic acid cycle (GO:0006099, p < 0.01, Fig. 1, Additional file 2). In *S. cerevisiae*, Arfs play crucial roles in vesicle trafficking, cytoskeletal organisation, mitochondrial homeostasis, mitotic growth, amongst other fundamental processes, indicating the function of these regulators is indeed diverse [22, 29]. There have also been observations that respiration may be impacted by Arf GTPases [30]. Based on analysis of co-expression data, we therefore hypothesised that modulation of *A. niger ageB*, *secG*, and *geaB* expression may impact titres of secreted protein and, additionally, titres of secreted citric acid.

**Table 1:** Genes belonging to *secG*, *geaB*, or *ageB* subnetworks (Fig. 1) with a predicted functional role at either the Golgi or during the TCA cycle

GO	Name	ORF code	Predicted function
Golgi associated vesicle membrane (GO:0030660)	*copA*	An16g02460	COPI complex, alpha subunit
	*sec26*	An08g03270	COPI complex, beta subunit
	*sec21*	An07g06030	COPI complex, gamma subunit
	*sec27*	An02g05870	COPI complex, beta subunit
	*sec23*	An01g04730	COPII complex subunit
	*ret2*	An01g14250	COPI complex, delta subunit
	*apm1*	An07g03200	Mu1-like medium subunit of the AP-1 complex; involved in clathrin-dependent Golgi protein sorting
	*aplD*	An01g02600	Gamma-adaptin; large subunit of the AP-1 complex; involved in clathrin-dependent Golgi protein sorting
Golgi vesicle budding (GO:0048194)	*glo3*	An16g05370	ADP-ribosylation factor GTPase activating protein; involved in ER-Golgi transport
	*sec13*	An04g00360	COPII complex subunit
	*sec16*	An15g01520	COPII vesicle coat protein required for ER transport vesicle budding as well as in COPII-mediated ER-to-Golgi traffic; interacts with Sec23p, Sec24p and Sec31p
Tricarboxylic acid cycle (GO:0006099)	*kgdA*	An04g04750	Oxoglutarate dehydrogenase
	*mdhA*	An07g02160	Mitochondrial malate dehydrogenase
	*idh1*	An18g06760	Isocitrate dehydrogenase
	*idh2*	An08g05580	Isocitrate dehydrogenase
	*ico2*	An09g03870	Aconitate hydratase
	*citA*	An09g06680	Citrate synthase
	*aclA*	An11g00530	Cytoplasmic ATP-citrate lyase
	*kgd2*	An11g11280	Dihydrolipoamide S-succinyl transferase
	*fumR*	An12g07850	Fumarate hydratase
	*aclB*	An11g00510	ATP:citrate oxaloacetate lyase

Predicted functions and gene names were taken from either AspDB [28] or SGD [31]

### Expression of *A. niger ageB*, *secG*, and *geaB* impact filamentous growth on solid media

Prior to conducting gene functional experiments, we conducted in silico analyses of predicted amino acid sequences to provide evidence that genes An11g02650 (*ageB*), An07g02190 (*secG*), or An18g02490 (*geaB*) do indeed encode ArfA GAP or GEFs. Sequences from key domains of *S. cerevisiae* orthologues [31] were thus aligned with each respective *A. niger* protein. The yeast Age1 protein contains an Arf GTPase activating domain of 114 amino acids which demonstrated 51.8% sequence conservation with a domain encoded by An11g02650 (Additional file 3). Similarly, the yeast Sec7 protein contains an ~ 200 amino acid domain which mediates ARF GEF activity [32], which was 55.5% conserved with the predicted An07g02190 protein. Additionally, yeast Gea2 also contains a ~ 200 amino acid Sec7 domain, which was 38.8% conserved with the putative *A. niger* GeaB protein encoded by gene An18g02490 (Additional file 3). Conservation of key protein domains with yeast orthologues is consistent with the function of An11g02650 as an ArfA GAP, and An07g02190/An18g02490 as ArfA GEFs.

In order to probe the role of these genes in protein and citric acid fermentation, we generated conditional expression isolates in which a Tet-on cassette was placed immediately upstream of the gene of interest [25, 33]. This cassette is titratable by addition of the stable tetracycline derivative doxycycline (Dox) to growth media, has undetectable levels of basal expression in the absence of induction, and addition of 20 µg/ml Dox enables expression above that of the *A. niger* glucoamylase gene commonly used for over-expression studies [10, 25, 33]. Thus, the titratable expression of the Tet-on cassette enabled the analysis of null, intermediate, and over-expression phenotypes in a single strain. *A. niger* isolates were generated by a recently developed genome editing protocol, with guide RNA expression driven by the 5S rRNA gene as promoter [24]. PCR verified isolates were recovered for *ageB* (strains TC5.5/TC5.6), *secG* (strains TC4.4/TC4.5), and *geaB* (strain TC6.1). Note that while numerous transformants were recovered for *secG* conditional expression strains, over 5 transformations only generated two and one clone for *ageB* and *geaB*, respectively, which presumably was due to the poor growth of primary transformants in these isolates (see below). In this study, strains TC4.4/TC4.5 and TC5.5/TC5.6 were assayed in parallel for all experiments. However, these isogenic strains performed highly comparably and did not display any significant differences in any of the assays, and consequently for clarity we only report data for isolates TC4.4 and TC5.5 unless otherwise stated.

**Fig. 2:**
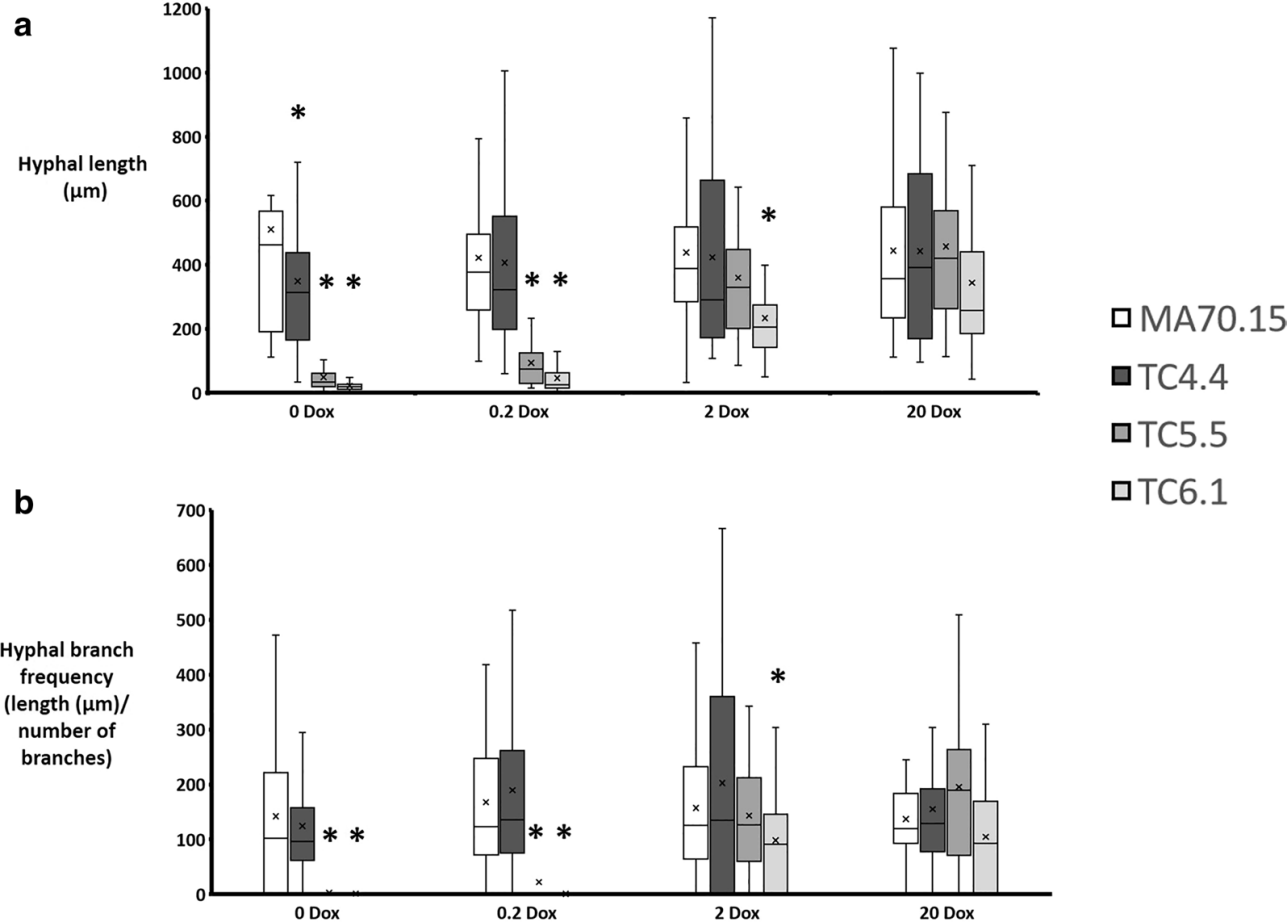
Quantitative analysis of early hyphal growth in conditional expression mutants following titration of gene expression. 1 × 10^4^ spores/ml were inoculated in 10 µl volumes onto MM supplemented with various concentrations of Dox (μg/ml). Plates were incubated at 30 °C in the dark for 18 h. Box whisker plots depicting hyphal length (µm, A) and branching rate (length µm/total number of branches, B) are given. Experiments were technically triplicated. Note that *secG* (TC4.5) and *ageB* (TC5.6) mutants performed comparably to their isogenic comparator, and are omitted from this figure for clarity. Asterisk indicate significant differences between progenitor control (MA70.15) and conditional expression isolates using a Student‘s *t* test. A minimum of 30 hyphae were analysed per strain/condition

We firstly quantified the impact of *ageB*, *secG*, or *geaB* expression levels on *A. niger* spore germination and hyphal development. Spores were inoculated on solid MM, grown for 18 h at 30 °C, and length and branch frequency quantified using ImageJ2/Fiji ([34], Fig. 2). When Dox was omitted from growth media, *secG* in isolate TC4.4 resulted in a minor, yet statistically significant reduction in hyphal length when compared to the progenitor isolate MA70.15. Hyphal branch rates however were not impacted in this mutant. Under all other Dox concentrations, hyphal length and branching in the *secG* mutant was comparable to the control (Fig. 2). These data suggest that *secG* has only a minor impact on early hyphal development in *A. niger*. In contrast, gene expression using 0 and 0.2 µg/ml Dox resulted in highly defective germling development in both the *ageB* (TC5.5) and the *geaB* (TC6.1) mutants. Under these conditions, these isolates produced short germlings in which branching was rarely observed (Fig. 2). Titration of *ageB* gene expression in isolate TC5.5 using 2 or 20 µg/ml Dox resulted in both length and branch rates which were comparable to that of the progenitor strain (Fig. 2). Isolate TC6.1 required expression of 20 µg/ml Dox to have comparable length/branching as the progenitor control. These data suggest *ageB* and *geaB* are dispensable for polarity establishment and thus germination, yet are important for polarity maintenance and branching of young hyphae.

### *A. niger ageB*, *secG*, or *geaB* expression is required for colony formation, sporulation, and resistance to oxidative and elevated temperature stress

**Fig. 3:**
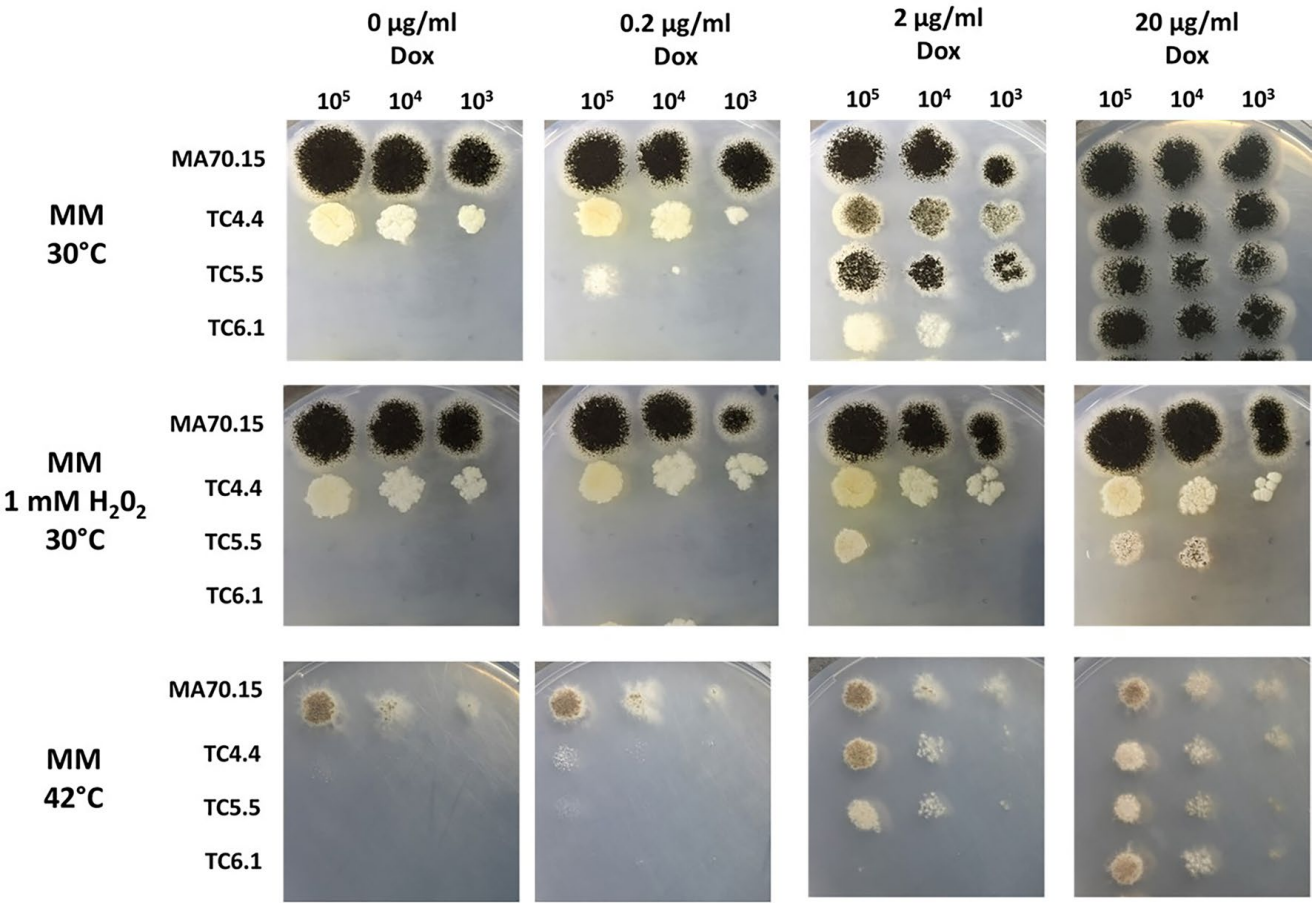
Phenotypic screening of conditional expression mutants reveals growth defects and sensitivity to abiotic stress. 1 × 10^5^–1 × 10^3^ spores/ml were inoculated in 10 µl volumes onto MM supplemented with various concentrations of Dox. Plates were incubated at indicated temperature in the dark, and images captured after 3 days (30 °C) or 6 days (MM 42 °C). Representative images are shown for technically triplicated experiments. Plates were supplemented with hydrogen peroxide as indicated

Phenotypic screens were conducted on solid agar supplemented with 0, 0.2, 2 and 20 µg/ml Dox in order to model null, low, intermediate, and over-expression respectively. Expression using 0 and 0.2 µg/ml Dox revealed severe defects in growth for all isolates, with *secG* conditional expression strain TC4.4 revealing compact, aconidial colonies (Fig. 3). Under 0 µg/ml Dox, *ageB* and *geaB* conditional expression mutants TC5.5 and TC6.1 grew at a level that was only detectable by microscopic inspection (Fig. 3 and data not shown), confirming the product of these genes are essential for colony growth. Defects in growth were titratable using this assay, as all strains resembled that of the progenitor isolate when media was supplemented with 20 µg/ml doxycycline, providing strong evidence that growth defects were due to mis-expression of the predicted GEF or GAP (Fig. 3). The observed morphological defects when media was supplemented with 0 or 0.2 µg/ml Dox occurred independently of glucose concentration (0.1%, 10%) or carbon source (1% fructose, data not shown).

In order to provide evidence that the growth defects in conditional expression isolates were due to defects in the *A. niger* secretory pathway, we supplemented media with sub-lethal concentrations of hydrogen peroxide, or incubated plates at elevated temperature (42 °C), known to provoke protein folding stress at the ER and Golgi [35, 36]. All isolates were sensitive to sub-lethal oxidative stress as well as elevated temperature (Fig. 3). These data support the role of the predicted ArfA GEF and GAP proteins in regulating the secretory pathway in *A. niger*.

Previously, we have demonstrated loss-of-function of *arfA* results in sensitivity to growth of 1% starch, which is likely due to an inability to secrete the extracellular glucoamylase GlaA [21]. We did not observe such a defect in the loss-of-function strains in the current study (data not shown). Moreover, *arfA* loss-of-function strains were previously shown to be sensitive to chitin-based cell wall perturbation due to defects in supply of cell wall building enzymes to the hyphal apex. In contrast, sensitivity to calcofluor white based chitin stress was also not observed in strains generated in this study (data not shown). These data suggest that defects in secretion may not be as severe when modulating expression of the predicted ArfA GEFs or GAP when compared to that of ArfA [21].

### Expression of *ageB* and *geaB*, but not *secG*, have major impacts on *A. niger* macromorphology and extracellular protein titres during submerged growth

**Fig. 4:**
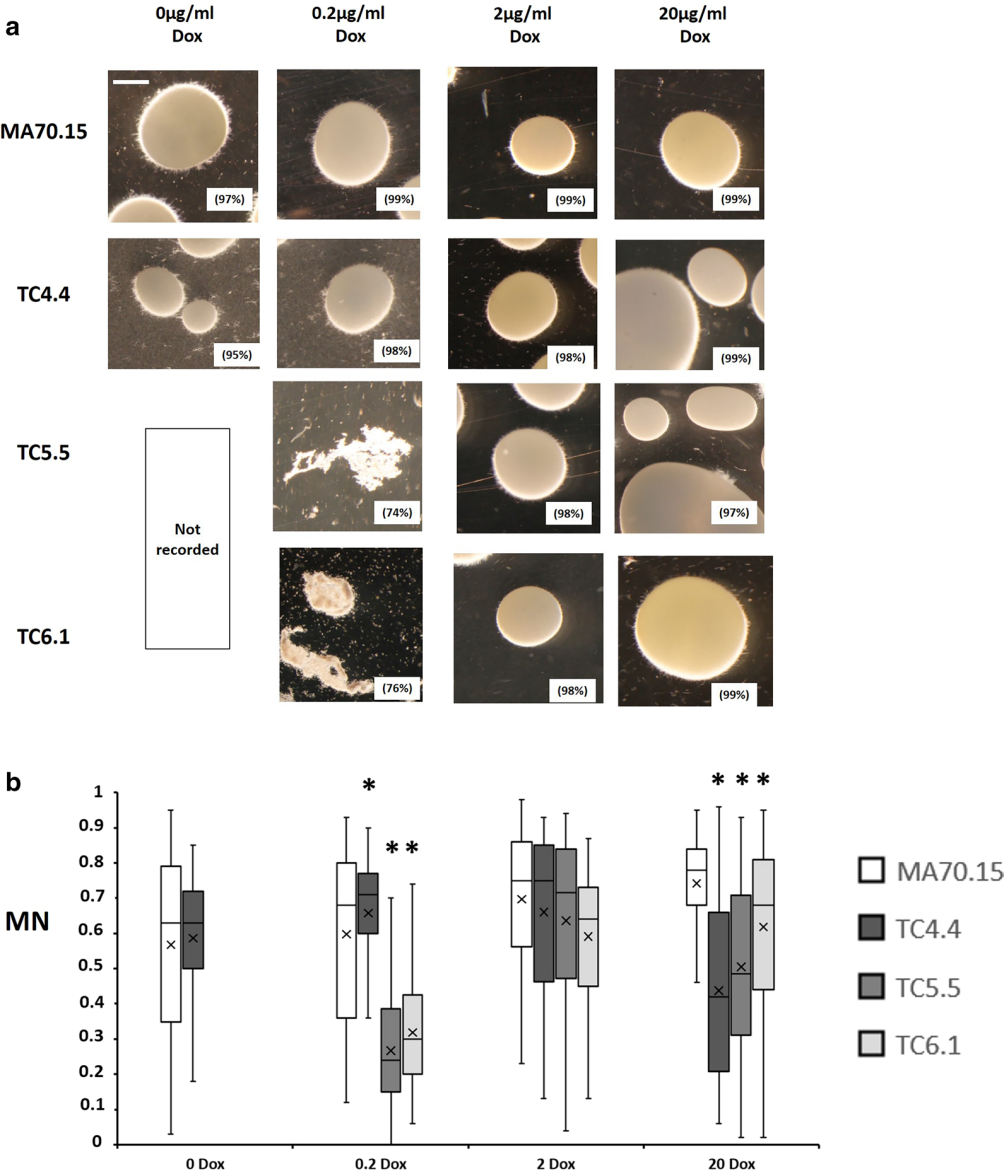
Representative images and quantitative analysis of conditional expression mutant macromorphology during submerged growth in minimal media. To model protein fermentation conditions, 1 × 10^6^ spores/ml of conditional expression mutants and progenitor control (MA70.15) were inoculated in 20 ml MM with 5% glucose as carbon source and supplemented with various concentrations of Dox. Cultures were grown at 220 RPM, 30 °C, for 72 h. **a** Representative images are depicted for triplicated experiments each consisting of duplicate replicates. Pelleted morphologies (any fungal structure > 500 µm^2^ area) are reported as a function of the total fungal area measured during image analysis, and are indicated as a percentage in parenthesis. Scale bar in the top left panel is 1 mm. **b** Shake flask cultures were quantitatively analysed using the MPD image analysis pipeline [37]. Reported are box whisker plots for pellet morphology number (MN). Crosses depict average values. Pairwise Student‘s *t*-tests were conducted between conditional expression mutant relative to the MA70.15 control at respective Dox concentrations. *p* values are indicated as (< 0.05, *)

In order to assess the role of *secG, ageB,* and *geaB* expression on submerged growth and extracellular protein titres, conditional expression mutants and progenitor control were cultured in liquid media conventionally used to achieve high protein production (MM, 5% glucose, 30 °C, pH 5.6). Cultivation media were supplemented with 0, 0.2, 2 and 20 µg/ml Dox, with the exception of strains TC5.5 (*ageB*) and TC6.1 (*geaB*), for which 0 µg/ml Dox concentrations were omitted due these genes being essential (Fig. 3). Representative images of pellet morphology at the end of shake flask cultivations are shown in Fig. 4a and a summary of growth phenotypes given in Table 2. Additionally, pellet Euclidian parameters (maximum diameter, area, solidity, and aspect ratio, see methods) were quantified using the automated Morphology of Pelleted and Dispersed growth (MPD) image analysis pipeline [37] and used to determine the dimensionless morphology number (MN [38]), which generates a value between 0 (a theoretical one-dimensional line) and 1 (a perfect round sphere, Fig. 4b).

**Table 2:** Summary of phenotypic consequences following expression of predicted ArfA GEFs or GAP using 0 or 0.2 µg/ml Dox

Phenotype	Mutant strain/Dox concentration		
	*secG* (0 µg/ml Dox)	*geaB* (0.2 µg/ml Dox)	*ageB* (0.2 µg/ml Dox)
Filamentous growth	−	+++	+++
Colony development	+	+++	+++
Pellet formation during protein production	−	+++	+++
Extracellular protein	−	+++	+++
Pellet formation during organic acid production	+++	+	+
Extracellular citric acid	+++	+++	+++

**Fig. 5:**
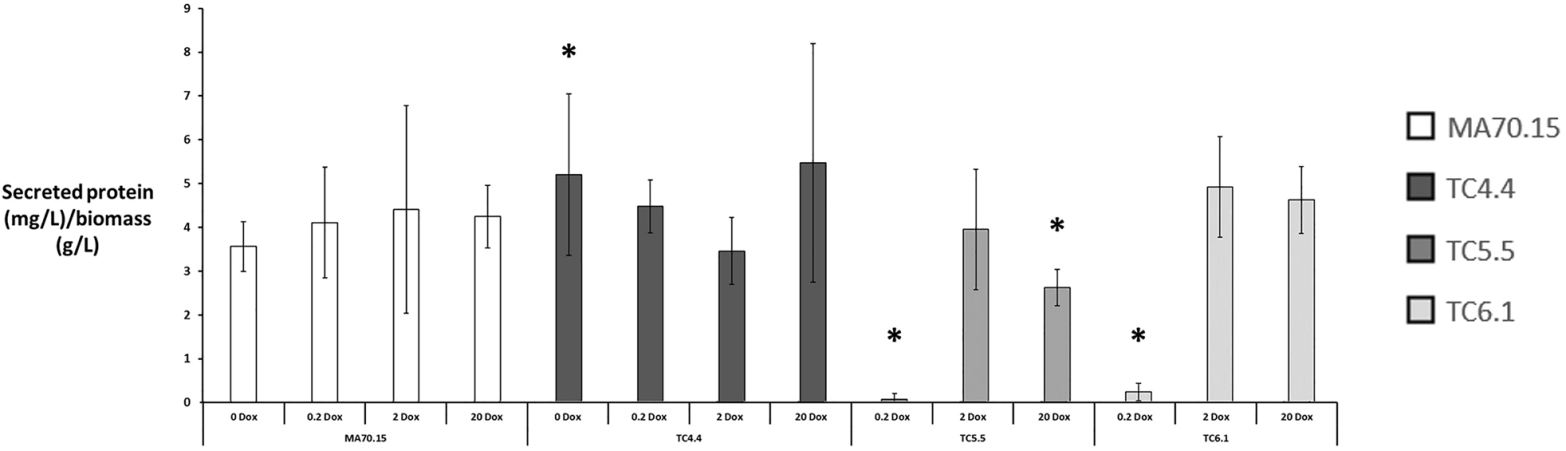
*A. niger* genes *geaB* and *ageB*, but not *secG,* have critical impacts on protein titres during submerged culture. Total protein in supernatant was determined using a Bradford assay, which was normalised to fungal dry weight. Pairwise Student‘s *t*-tests were conducted between conditional expression mutant relative to the MA70.15 control at respective Dox concentrations (μg/ml). *p* values are indicated as (< 0.05, *). Note that mutants TC4.5 and TC5.6 performed comparably to their isogenic comparator, and are omitted from this figure for clarity

**Fig. 6:**
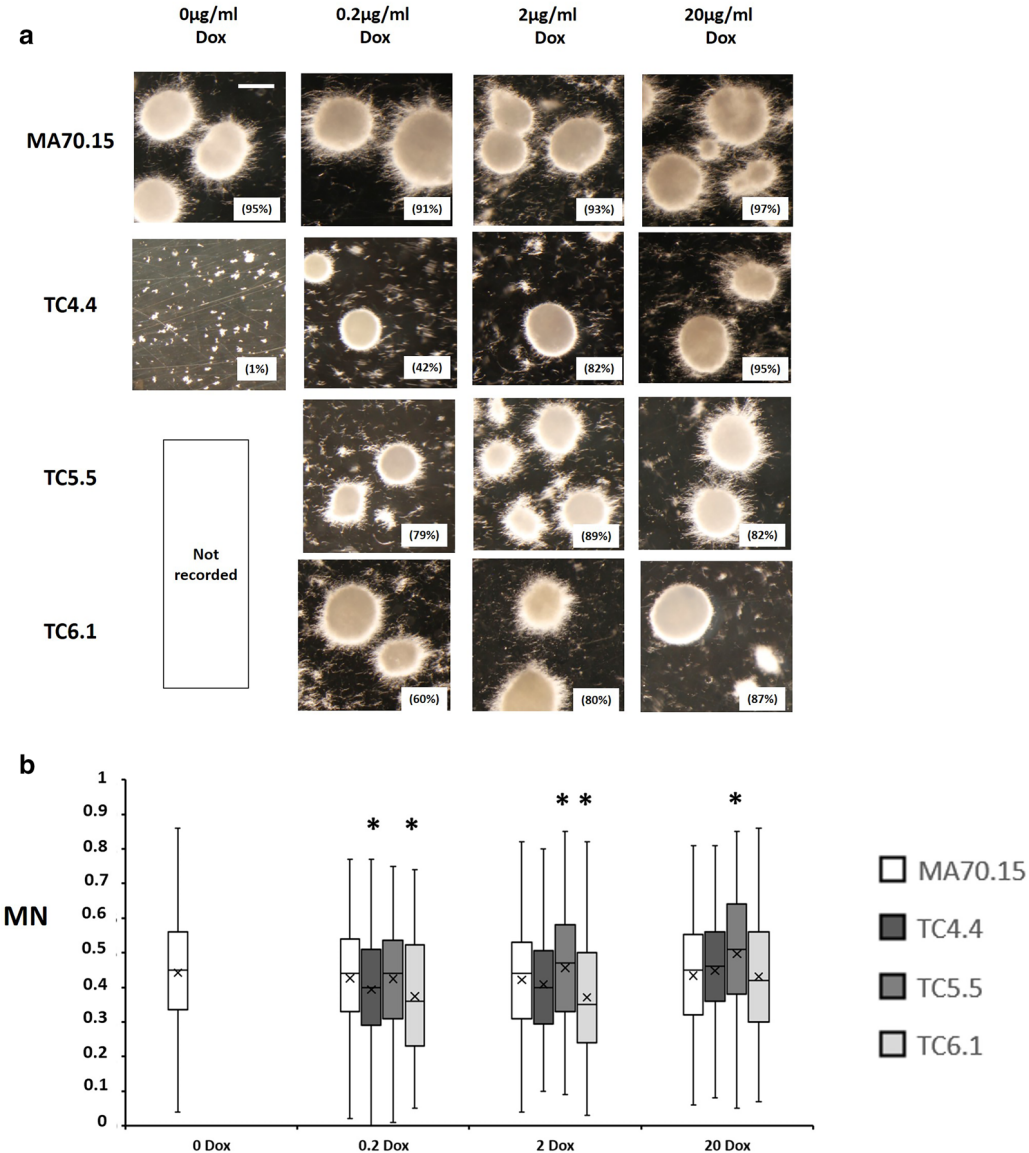
Representative images and quantitative analysis of conditional expression mutant macromorphology during submerged growth in citric acid production media. 1 × 10^5^ spores/ml of each isolate were inoculated in organic acid production medium CitACM with 10% sucrose as carbon source. Cultures were grown at 220 RPM, 34 °C, for 96 h (see “Methods” section for full conditions). Representative images are depicted for triplicated experiments each consisting of duplicate replicates (**a**). Pelleted morphologies (any fungal structure > 500 µm^2^ area) are reported as a function of the total fungal area measured during image analysis, and are indicated as a percentage in parenthesis. Scale bar in the top left panel is 1 mm. Shake flask cultures were quantitatively analysed (**b**) using the MPD image analysis pipeline as described in Fig. 4b. Note that pelleted morphologies were almost entirely absent in the *secG* mutant TC4.4 0 µg/ml Dox during growth in organic acid production medium

**Fig. 7:**
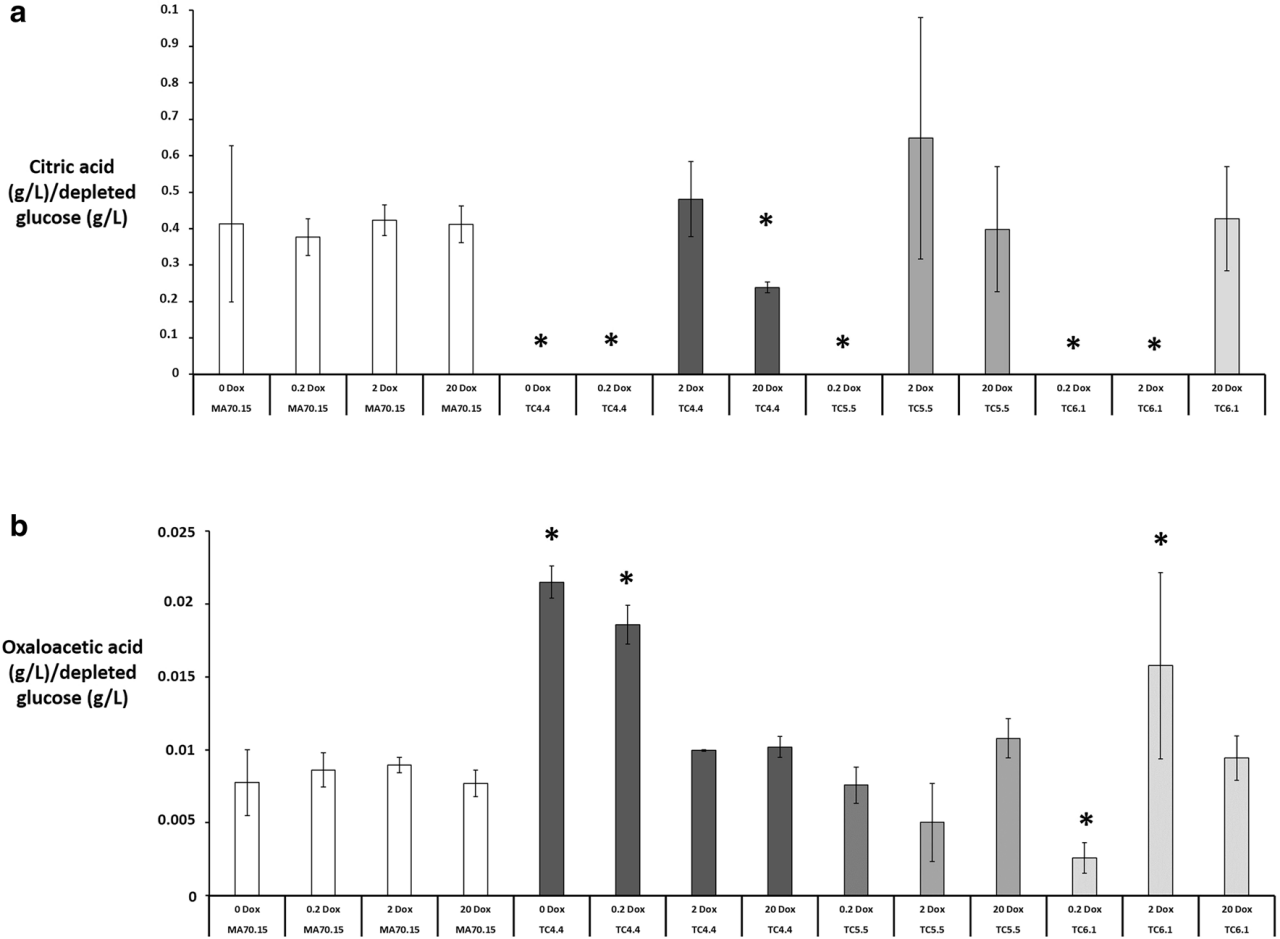
*A. niger* genes *secG*, *ageB*, and *geaB* have critical impacts on organic acid titres during submerged culture. From organic acid production medium in shake flask cultures, secreted citric acid and oxaloacetate were quantified using HPLC, and normalised to depleted glucose. Pairwise Student‘s *t*-tests were conducted between conditional expression mutant relative to the MA70.15 control at respective Dox concentrations (μg/ml). *p* values are indicated as (< 0.05, *). Note that mutants TC4.5 and TC5.6 performed comparably to their isogenic comparator, and are omitted from this figure for clarity

Phenotypic deviations from the progenitor are indicated as dispensable (−), moderate (+) or essential (+++). For quantification, please see Figs. 2, 3, 4, 5, 6 and 7

Under all Dox concentrations, the progenitor control produced approximately spherical pellets several millimetres in diameter, with quantitative image analysis confirming that > 96% of fungal growth consisted of pellets (Fig. 4a). Mutants TC5.5 and TC6.1 displayed irregularly shaped aggregates and elevated dispersed mycelia under 0.2 µg/ml Dox (Fig. 4a, b) suggesting *geaB* or *ageB* expression is important for pellet formation during protein fermentation respectively. Addition of 2 µg/ml Dox to strains TC5.5 or TC6.1 in this growth media resulted in near wild-type pelleted macromorphology. In contrast, *secG* mutants were able to produce pellets under 0 µg/ml Dox, although these were slightly smaller in diameter when compared to MA70.15 (Fig. 4). Interestingly, expression of either *secG*, *ageB*, or *geaB* using 20 µg/ml Dox under protein production conditions resulted in two distinct sizes of pellets, which were either significantly larger or smaller that the progenitor control, leading to modified MN under these conditions (Fig. 4).

Next, we measured total extracellular protein in culture supernatants using a Bradford assay (Fig. 5). These data suggest *A. niger secG* expression was dispensable for protein secretion, as media supplemented with 0.2, 2 and 20 µg/ml Dox displayed comparable extracellular total protein to that of the progenitor control (Fig. 6a). It should be noted that a small, but statistically significant, elevation in total protein titres was observed for *secG* mutants TC4.4/TC4.5 under 0 µg/ml Dox when compared to the progenitor (Fig. 5 and data not shown). Culture supernatants from mutants TC5.5 and TC6.1 were drastically reduced in extracellular protein when grown under 0.2 µg/ml Dox, but displayed comparable levels to the control with 2 µg/ml Dox (Fig. 5). These data are consistent with the severe morphological defects in these isolates under 0.2 µg/ml Dox when grown in MM (Fig. 4). It should be noted that expression using 20 µg/ml Dox in isolate TC5.5 resulted in a statistically significant reduction in extracellular protein relative to the control (Fig. 5), indicating that elevated expression of *ageB* also perturbs protein secretion. Taken together, we conclude that GeaB and AgeB are important for the development of macromorphological structures and protein secretion during shake flask culture, whereas SecG is largely dispensable.

### *ageB*, *secG*, and *geaB* expression impacts organic acid titres in culture media during *A. niger* submerged growth

Next, we assessed colony macromorphology and product titres under conditions routinely used to achieve high organic acid production (CitACM, 10% sucrose, 34 °C, pH 2.5). We observed a slight elevation in hyphal fragments in CitACM (Fig. 6a) when compared to protein media (Fig. 4a), and more extensive hyphal growth at the pellet periphery, which is consistent with previous data [37]. Additionally, all conditional expression mutants tended to display higher levels of filamentous growth during citric acid fermentation when compared to the control, which was especially pronounced at lower Dox concentrations (Fig. 6).

With regards to individual gene function, *secG* mutants were almost completely unable to form any pellets in citric acid media without Dox, with ~ 99% of fungal morphologies from mutants TC4.4 qualified as dispersed mycelia and hyphal fragments (Fig. 6). Addition of 0.2 and 2 µg/ml Dox to *secG* mutant culture resulted in the formation of pellets, although more hyphal fragments were still observed when compared to the progenitor control (Fig. 6). These data demonstrate that expression of *secG* is critical for pellet formation during citric acid fermentation (Table 2).

In contrast, expression of both *geaB* and *ageB* using 0.2 µg/ml Dox resulted in a comparable pellet morphology in mutants TC5.5 and TC6.1 when compared to the progenitor control, although elevated dispersed morphology was also observed (Fig. 6a). Increased expression of either gene using 2 or 20 µg/ml resulted in higher percentage of pellet formation when compared to 0.2 µg/ml Dox (Fig. 6), so that growth was broadly comparable to the progenitor control.

Next, we interrogated citric acid and oxaloacetic acid titres in supernatants during submerged cultivation by HPLC. Citric acid was absent in the TC4.4 *secG* mutant culture under 0 and 0.2 µg/ml Dox (Fig. 7a). Moreover, we observed reduced citric acid concentrations in *secG* mutants relative to the progenitor control using 20 µg/ml Dox, which is consistent with the hypothesis that expression of *secG* is tightly linked with *A. niger* citric acid titres. Additionally, we found a clear increase in oxaloacetic acid in supernatants of *secG* mutants under 0 and 0.2 µg/ml Dox relative to control (Fig. 7b).

Similarly, we did not detect citric acid in cultures of the *geaB* mutant under 0.2 and 2 µg/ml Dox (Fig. 7a). However, levels comparable to the progenitor were observed under 20 µg/ml Dox (Fig. 7a). Combined with the organic acid profile of the *secG* mutant, these data suggest that expression of both *secG* and *geaB*, and thus ArfA GEFs in general, impacts citric acid titres during submerged growth. Interestingly the *geaB* mutant resulted in lowered oxaloacetate (0.2 µg/ml Dox), elevated oxaloacetate (2 µg/ml Dox) and wild-type levels of oxaloacetate (20 µg/ml Dox, Fig. 7b), providing further evidence that both ArfA GEFs play an important role during fermentation of this metabolite.

Citric acid was not detected in supernatants of the *ageB* mutant under conditions 0.2 µg/ml Dox, but was present at concentrations comparable to the progenitor control under 2 and 20 µg/ml Dox, supporting the notion that this predicted ArfA GAP impacts organic acid titres. It should be noted that oxaloacetate concentrations were comparable to the control under all conditions tested for *ageB* mutants (Fig. 7b), implying that the importance of the predicted ArfA GEFs SecG and GeaB for high citric acid titres is more pronounced than the importance of the predicted ArfA GAP AgeB.

Given the crucial role of mitochondria in citric acid production, we reasoned reduced titres of this organic acid in growth media (Fig. 7a) may be due to aberrant localisation of these organelles in conditional expression mutants. However, staining of germlings using MitoTracker (Thermo-Fisher) revealed comparable mitochondrial localisation in mutants TC4.4, TC5.5, and TC6.1 relative to the MA70.15 control (Additional file 4).

### Protein and citric acid titres are correlated in *ageB*, *secG*, and *geaB* conditional expression mutants

**Fig. 8:**
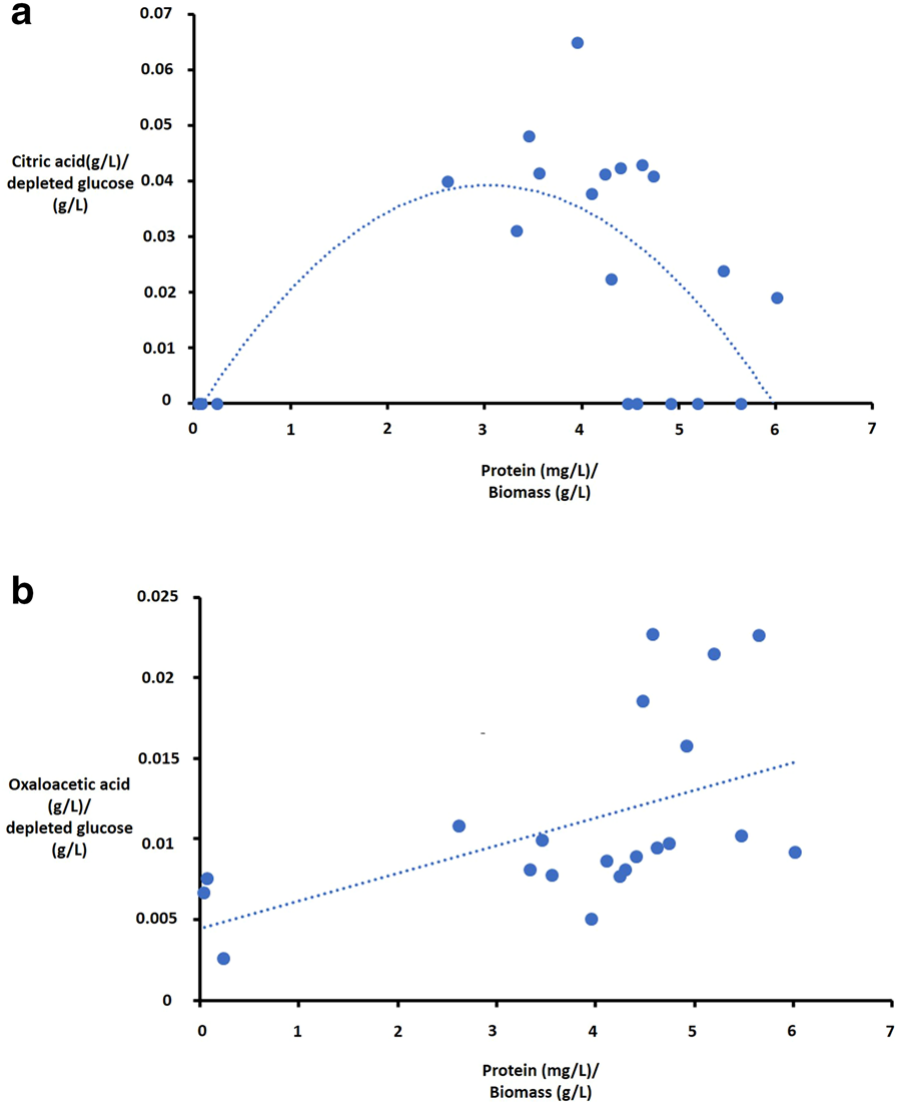
Plotting protein and citric acid/oxaloacetate titres reveals a possible correlation between these processes amongst *A. niger* GEF and GAP conditional expression mutants. Total extracellular protein (mg/g biomass) at various Dox concentrations in progenitor control (MA70.15) and all conditional expression mutants TC4.4, TC4.5, TC5.5, TC5.6 and TC6.1 were plotted as a function citric acid or oxaloacetic acid concentrations in culture supernatant (g/g depleted glucose). Trend lines were estimated for citric acid/protein titres using polynomial of degree 2 (**a**) or linear fit for oxaloacetic acid/protein titres (**b**). R^2^ values for these trend lines were 0.44 and 0.27, respectively

Given that each mutant displayed various concentrations of organic acid and total protein in media supernatants, we reasoned that extracellular titres of these molecules might be correlated in *A. niger*. Consequently, we plotted average extracellular protein against average citric acid or oxaloacetate titres for each strain/Dox concentration (Fig. 8). Notably, a curve correlation was obtained for total protein and citric acid titres for the strains tested in this study (Fig. 8a). Additionally, there was a weak, linear positive correlation between protein and oxaloacetate abundance (Fig. 8b). Taken together, data presented in this study suggest that *A. niger* SecG, GeaB, and AgeB associated processes are crucial for product titres and macromorphologies of *A. niger*.

## Discussion

Filamentous fungi have been used for over a century for the production of useful molecules, including organic acids, enzymes, and secondary metabolites [1, 4]. Improving product titres is a major aim of fungal biotechnologists, which will ultimately enable the efficient contribution of these cell factories towards the global bioeconomy [2].

We have recently demonstrated that co-expression networks are a powerful resource for assigning gene function in *A. niger* [10]. In this previous work, co-expression sub-networks were used to identify two novel transcriptional regulators of secondary metabolite biosynthesis (MjkA, MjkB), and functional analysis of these genes using the Tet-on system enabled awakening of the natural product biosynthesis reservoir of *A. niger* [10]. In the current study, we used this genome-wide co-expression resource to identify genes which impact primary metabolism and growth, specifically protein and organic acid titres. The TCA cycle/Golgi co-expression network generated in this study consisted of 259 candidates which were co-expressed with 6 target genes, including those encoding the predicted ArfA GEFs SecG/GeaB and GAP AgeB (Fig. 1). We have recently demonstrated that *A. niger* ArfA controls protein secretion, morphology, and the position of the actin ring at the hyphal apex [21]. It should be noted that the *arfA* gene [21] was absent from the 259 gene network, which would be consistent with its main regulation at the protein level, i.e. GTP activation or GDP deactivation via ArfA GEFs and GAPs. We thus reasoned that titratable expression of GEF and GAP regulators might offer a unique opportunity to concomitantly modulate protein secretion, organic acid titres, and the macromorphology of *A. niger*. Indeed, there has been much recent interest in morphological control of fungal production isolates during submerged growth, as this has critical implications for product titres and may optimise fermentation due to improved rheological behaviour of *A. niger* during bioreactor cultivations [1, 39–42].

Growth assays on solid media demonstrated that *geaB* and *ageB* genes were essential. These data highlight the utility of CRISPR–Cas9 Tet-on promoter replacement approach [24, 25], as conventional deletion strategies would not enable functional analysis of these genes. In terms of gene function, growth on solid media have demonstrated that *secG* plays a minor role in germination and early hyphal growth but is required for colony development and conidiation. These data suggest that this gene likely controls hyphal growth at later stages than those assessed in the microscopic growth assay used in this study (MM, 30 °C, 18 h).

With regards to submerged growth, media composition was critical for strain performance and product titres (Figs. 4, 5, 6, 7), and we observed drastic differences in either pellet formation and/or productivity amongst strains, which was remarkable (Table 2). The fact that both *ageB* and *geaB* drastically impact macromorphology and extracellular protein in shake flask culture was consistent with the well-established role of the Golgi in delivery of vesicles to the hyphal apex [21]. It was surprising that *secG* was dispensable for these processes, which might be explained by functional redundancy of this gene, with *geaB* likely encoding most ArfA GEF activity in *A. niger*. This hypothesis is consistent with the phenotypic observations of *secG* and *geaB* mutants during growth on solid media without Dox (Figs. 2, 3). From a biotechnological perspective, the increase in protein production following loss of *secG* expression (Fig. 5) may offer future avenues for maximizing titres in enzyme fermentation. However, it should be noted that the increase was small (Fig. 5) and requires validation under bioreactor cultivations prior to any firm conclusions about the validity of this approach.

The drastic reduction/absence of citric acid in the supernatant of mutant cultures under 0/0.2/20 µg/ml Dox (*secG* mutants), 0.2/2 µg/ml Dox (*geaB* mutant) or 0.2 µg/ml Dox (*ageB* mutants) strongly suggests that ArfA dependent processes are required for citric acid production and/or secretion. This work thus lays the groundwork for future studies aiming to understand the function and precise spatial and temporal recruitment of the predicted regulatory proteins to ArfA, as they have not been studied so far in *A. niger*. Still, their high sequence homology to the well-studied *S. cerevisiae* orthologs *sec7*, *gea2*, and *age2* as well as the functional conservation of GTPase regulation via GAPs and GEFs in all eukaryotic kingdoms, make it reasonable to speculate that *secG*, *geaB*, and *ageB* encode ArfA regulators in *A. niger*.

What might be the mechanistic basis for reduced citric acid in culture supernatants following reduced ArfA GEF or GAP expression? As we measured secreted products in the supernatant only, the effect of *secG*, *geaB*, and *ageB* expression on protein and citric acid titres might occur on different levels. Our data are consistent with either (i) dysfunctional trafficking of organic acid transporter(s) to the plasma membrane; (ii) defective delivery of TCA biosynthetic enzymes to the mitochondria; (iii) inhibition of the location/function of the citric acid synthase CitA; (iv) altered citric acid-malate shuttle or (v) a combination of these factors. Interestingly, the citrate transporter in *A. niger*, CexA, has recently been discovered [43], and it is possible that transport of this protein to the plasma membrane is dependent on ArfA GEF/GAP function. Although we did not detect any differences in mitochondrial localisation in conditional expression mutants (Additional file 4), the ER-mitochondria encounter structure (ERMES), has recently been functionally analysed in *A. fumigatus* [44]). ERMES tethers the mitochondria and endoplasmic reticulum, and may be required for transport of enzymes to the mitochondria. Indeed, the small GTPase Sar1 regulates ER-mitochondrial contact site size in *S. cerevisiae* [45]. It is interesting to speculate that ERMES function, and transport of TCA cycle enzymes to the mitochondria, might be SecG, GeaB, AgeB (and thus ArfA) dependent in *A. niger*, although testing this hypothesis is outside the scope of this study.

It is also interesting that *secG* and *geaB* mutants displayed an increased titres of the citric acid precursor oxaloacetate at various Dox concentrations (Fig. 7). It is possible therefore that transporters for this molecule are also dependent on SecG/GeaB, and ultimately ArfA activation. Another possible hypothesis is that ArfA GEFs may indirectly affect localisation and/or function of the CitA enzyme. Based on HPLC data, it seems that SecG plays a predominant role in the elevated oxaloacetate concentrations, as this was observed in both 0 and 0.2 µg/ml Dox, as opposed to a single condition for *geaB* (2 µg/ml Dox).

With regards to the connection of macromorphological development and citric acid fermentation, our data on the phenotypes associated with *secG* loss-of-function are consistent with previous studies, which concluded that pellet formation is necessary for citric acid fermentation [1, 46]. However, our data suggest that two refinements of this notion are necessary: firstly, while pellets are required for citric acid production and/or secretion, other organic acids (e.g. oxaloacetate) can be detected in cultures consisting of dispersed mycelia (e.g. Figs. 6, 7, see *secG* mutant, 0 µg/ml Dox). Secondly, pellet formation and citric acid titres can be decoupled, as pelleted morphologies in *secG* (0.2 µg/ml Dox), *geaB* (0.2, 2 µg/ml Dox) and *ageB* (0.2 µg/ml Dox) mutants have reduced citric acid in growth media. We suspect that crucial genes for morphological development, vesicle trafficking, mitochondrial function, and membrane transport are differentially expressed under these conditions (Table 1), which is something we will test with future transcriptomic, metabolomic and gene functional analyses in our laboratories.

Finally, putative correlations between protein/citric acid titres and protein/oxaloacetic acid titres (Fig. 8) support the hypothesis that these processes are indeed coupled in *A. niger*. These data are consistent with ^13^C metabolic flux analyses of glucoamylase hyper-secretion and wild-type strains, which demonstrated elevated protein secretion is associated with increased carbon flux to the oxidative pentose phosphate pathway and reduced flux through the TCA cycle [47]. In general, an inverse correlation between citric acid/protein production and cell growth has been reported for *A. niger* [48, 49]. In agreement, a high specific protein production rate is also achieved at relatively low growth rates in the protein cell factory *Trichoderma reesei* [50]. Therefore, our data are in agreement with these reports and suggest that the efficient development of filamentous fungi as microbial cell factories requires integrative genetic engineering approaches which couple different growth-related and product related processes.

## Conclusions

This study has demonstrated that harnessing the genome-wide gene co-expression network for *A. niger* is a powerful approach to uncover surprising links between so far independently considered processes in this cell factory. We demonstrate that expression of the predicted ArfA GEF and GAP encoding genes are prerequisites for fermentation of citric acid. Additionally, *geaB* and *ageB* expression underpins growth and product titres during protein fermentation. Our data suggest that concentrations of extracellular protein and organic acids are interconnected in *A. niger*, and that *secG*, *geaB* and *ageB* encoded products are likely an important nexus connecting *A. niger* primary metabolism. We suggest that future fungal strain engineering efforts should assess productivity from both protein and organic acid perspectives. Future experiments in our laboratories will reverse engineer the mechanistic basis of defective organic acid/protein fermentation/filamentous growth amongst the *secG*, *geaB*, and *ageB* mutants to gain fundamental insights into how these processes are integrated in *A. niger* on the molecular level.

## Methods

### Microbial strains

Fungal strains used in this study are given in Table 3. MA70.15 was used as progenitor isolate as this strain is deficient in the non-homologous end joining pathway, thus reducing ectopic integration events during transformation [51]. All bacterial plasmids were propagated in *Escherichia coli* DH5α using 100 µg/ml ampicillin as selection.

**Table 3:** Fungal strains used in this study

Name	Genotype	Reference
MA70.15	*kusA::amdS, pyrG*^−^	[51]
TC4.4	*kusA::amdS, pyrG*^−^*, PsecG::Tet*-*on*, *HygR*	This study
TC4.5	*kusA::amdS, pyrG*^−^*, PsecG::Tet*-*on*, *HygR*	This study
TC5.5	*kusA::amdS, pyrG*^−^*, PageB::Tet*-*on*, *HygR*	This study
TC5.6	*kusA::amdS, pyrG*^−^*, PageB::Tet*-*on*, *HygR*	This study
TC6.1	*kusA::amdS, pyrG*^−^*, PgeaB::Tet*-*on*, *HygR*	This study

### Media

Strains of *A. niger* were grown at 30 °C in minimal medium (MM) [21] or complete medium (CM), consisting of MM supplemented with 1% yeast extract and 0.5% casamino acids [21]. For citric acid production, CitACM liquid media consisted of 3 g/l (NH_4_)_2_SO_4_, 3 g/l NaNO_3_, 0.5 g/l yeast extract, and 100 g/l sucrose, with the pH adjusted to 2.5 using 100% HCl. All agar plates and liquid cultures were supplemented with 4 mM uridine.

### Co-expression analysis

The *A. niger* co-expression networks were analysed using FungiDB [6]. From 283 microarray experiments, co-expression networks for genes passing Spearman correlation coefficients above 0.5 were retrieved for query genes *citA* (An09g06680), *idh2* (An08g05580), *fumR* (An12g07850), *copA* (An16g02460), *sec13* (An04g00360) and *sec26* (An08g03270). Next, genes common to all 6 sub-networks were identified, giving 259 candidates. GO-enriched biological processes in this list were identified relative to the *A. niger* genome using default parameters in FungiDB, and those with Benjamini–Hochberg FDR corrected *p*-values above 0.05 were reported [6, 10]. Subsequently, sub-networks for genes encoding SecG (An07g02190), GeaB (An18g02490) and AgeB (An11g02650) were retrieved and enriched GO-terms amongst these subnetworks identified as described above.

### Alignment of putative GEF and GAP protein sequences

GEF and GAP domains from the model yeast *S. cerevisiae* were retrieved from the *Saccharomyces* genome database [31], whereas *A. niger* ORF sequences were downloaded from the Ensembl database [52]. Pairwise alignments were performed with JalView Version 2 [53] using default parameters.

### Molecular techniques

All molecular techniques were performed according to standard procedures described previously [21]. *A. niger* transformation and genomic DNA extraction were performed as described elsewhere [54], with 5–10 µg/ml doxycycline (Dox) added to primary transformation plates and sub-culture media. Primers used in this study are given in Additional file 5.

### Genome editing

CRISPR-mediated genome editing was conducted as described previously [37]. All plasmid sequences will be made available on reasonable request. Briefly, in order to design sgRNA with minimal chances of off-target cleavage, the 5′ UTR region of the *secG*, *geaB* and *ageB* genes were screened using the SsRNAcas9 Software against *A. niger* genome (Ensemble) to generate a 20 bp targeting locus [8, 55]. sgRNA oligos homologous to this target site were cloned into plasmid psgRNA6.0 [24] using BbsI. Generation of linear sgRNA constructs for *A. niger* transformation were generated by amplification using sequence verified *secG*, *geaB*, or *ageB* sg plasmids as template and primers M13F and M13R as previously described [24].

For donor DNA fragments necessary to insert the Tet-on cassette at the promoter regions, the Tet-on system [25] fused at the 3′ region of a hygromycin resistance cassette were amplified by PCR using primers containing 40 bp flanking regions to the promoter locus of either *secG*, *ageB* or *geaB* genes (primers are given in Additional file 5).

2 µg of the Cas9 encoding plasmid Cas9-Hyg (Zheng et al., in preparation) was co-transformed with 2 µg purified sgRNA and donor constructs into *A. niger* MA70.15 protoplasts as previously described [24]. Following selection (200 μg/ml hygromycin and 10 μg/ml Dox) and duplicate purification (200 μg/ml hygromycin and 5–10 μg/ml Dox) on MM supplemented, genomic DNA was extracted from putative transformants. Insertion of the donor cassette at the respective promoter region was confirmed by diagnostic PCR using verification primers (Additional files 5, 6). PCR confirmed *A. niger* transformants were stored in 25% v/v glycerol at − 80 °C. Isolates generated in this study were confirmed for single integration of the Tet-on cassette at the target locus using Southern blot analyses (Additional file 6).

### Growth quantification on solid media

Hyphal growth was measured on MM agar slices that were sufficiently thin (approx. 1 mm) for light microscopic analysis as described previously [37]. Briefly, 10 µl of 1 × 10^4^ spores/ml of mutant or control isolates were spotted in duplicate onto the agar slice, air dried, and incubated at 30 °C for 18 h after which images of fungal growth were captured using a Zeiss Axio Cam Mrc5 light microscope. All fungal morphologies were quantified for length and branch rate (length µm/number of branches) using ImageJ. Growth assays were repeated three times, with a minimum of 30 hyphae quantified per Dox concentration/strain.

### Phenotypic screens on solid media

Phenotypic screens were performed as described previously [37]. *A. niger* conidia were harvested from 5-day cultivated CM agar plates. For conditional expression mutants, agar was supplemented with 100 μg/ml hygromycin and 20 μg/ml Dox. Spores were harvested in sterile water, filtered through Miracloth, and washed twice by centrifugation in 30 ml sterile water. Defined spore titres of *A. niger* isolates were spotted in 10 µl volumes of ACM and MM agar plates, which were incubated for 7 days at 30 °C or 42 °C. Plates were inspected every 12 h and representative images were captured at indicated time points. Where specified, plates were supplemented with 1 mM H_2_O_2_. Phenotypic screens were conducted in technical triplicate.

### Protein production during submerged growth

Protein production in shake flasks was performed as previously described [21] with minor modifications. 1 × 10^6^ conidia/ml were inoculated in 20 ml MM supplemented with 5% glucose and different concentrations of Dox in 100 ml Erlenmeyer flasks, and cultivated at 30 °C and 220 rpm on a horizontal shaker for 72 h. 1 ml of filtered supernatant was flash frozen in liquid nitrogen for total protein quantification using a Bradford assay. Secreted protein was normalised to fungal dry weight. Duplicate replicates were conducted, each consisting of two cultures/strain/Dox concentration.

### HPLC analysis

For HPLC analysis of culture supernatant, 20 ml CitACM in 100 ml shake flasks were inoculated with 1 × 10^5^ spores/ml of the respective strains, which were incubated at 34 °C and 220 rpm for 96 h. Supernatants were isolated from cultures using filter paper, which were then centrifuged at room temperature for 12,000 rpm for 2 min. Total acid was estimated by titration, using 2 drops of 0.1% phenolphthalein as pH indicator, with 0.1429 M NaOH and a standard curve derived from citric acid. Depleted glucose was calculated using a Shandong Academy of Sciences SBA-40D bioanalyser. Next, supernatants were diluted in sterile distilled water either 1:2 or 1:5 depending on the estimated total acid volume. Samples were then boiled for 15 min at 100 °C, centrifuged a second time, and filtered through a 0.22 μm sterile filter membrane into a liquid phase HPLC tube. For HPLC analysis, mobile phase A consisted of ultrapure water filtered twice using a 0.22 um sterile filter. Mobile phase B consisted of 2.75 mM H_2_SO_4_ in ultrapure water which was also filtered twice using a 0.22 µm sterile filter. HPLC was conducted using a Shimadzu UFLC, equipped with Shimadzu LC-20AD infusion pump, SPD-20A UV detector, CTO-20A/AC column thermostat, SIL-20ACHT UFLC specification autosampler, and Shimadzu work station. The columns used were an Aminex HPX-87H (300 mm × 7.8 mm × 9 µm, BioRad) with guard column Shimadzu ODS-SP (5 µm, 3.0 mm × 10mm). Injection volumes were 10 µl, with a sample retention time of 25 min, a flow rate of 0.6 ml/min, UV detection wavelength of 210 nm, and column temperature of 50 °C. A minimum of 3 shake flask cultures were analyzed for each strain and Dox concentration.

### Quantitative assessment of submerged morphology

Cultures were analysed using an Olympus szx7 stereomicroscope connected to a Canon DS126251 camera as previously described [37]. For image capture, approximately 5 ml of culture volume was poured into a 25 ml petri dish, after which morphologies were gently agitated with a pipette tip to ensure pellets were physically separated. For each sample, triplicate images were captured from randomly selected regions of the petri dish. Images were captured on a black background with lighting from above to illuminate fungal pellets. Triplicate or duplicate replicates were conducted for growth in CitACM and MM respectively. Each replicate consisted of duplicate shake flasks per strain/Dox concentration.

Fungal morphologies were quantified in ImageJ/Fiji using the MPD plugin with default parameters [37]. Dispersed morphologies were defined as any fungal structure with an area < 500 µm^2^ and ≥ 95 µm^2^. Pellets were defined as any structure with an area ≥ 500 µm^2^. The following parameters were calculated for each fungal pellet: (i) area (µm^2^), (ii) Feret’s diameter (maximum diameter of each structure, µm), (iii) aspect ratio (maximum diameter/minimum diameter), (iv) solidity. Morphology numbers (MNs) were calculated as described earlier [38, 56]:

$$ Morphology \, Number = \frac{{2 \times \sqrt {Area} \times Solidity}}{{\sqrt \pi \times Feret^{\prime}s\;Diameter \times Aspect\;ratio}}. $$

### Determination of fungal biomass

To determine fungal biomass after imaging, cultures were filtered through triple layered muslin gauze, washed in sterile water, pat dried between paper towels, and added to pre-weighed falcon tubes. Biomass was incubated at 50–65 °C until dry (minimum of 24 h) after which dry weight was determined.

### Mitochondrial staining

Strains were inoculated into CitACM media and incubated as described above. After 8 h, early hyphae were collected from media by centrifugation, and resuspended in phosphate buffered saline (PBS). Cells were stained using 5 µm MitoTracker Green FM (Thermo Fisher, Germany) and incubated at 37 °C for 30 min. Samples were washed twice in PBS and imaged using an inverted TCS SP8 fluorescent microscope (Leica, Germany).

## Supplementary information

**Additional file 1.** Multi-gene co-expression network reveals transcriptional coupling of the Golgi and TCA cycle with ArfA GEFs and GAPs.

**Additional file 2.** Predictions derived from gene co-expression network analysis of *secG*, *ageB* and *geaB* connect *A. niger* GEFs and GAPs with protein secretion and the TCA.

**Additional file 3.** Conservation between yeast GEF and GAP domains with predicted *A. niger* AgeB, SecG, and GeaB proteins.

**Additional file 4.** Localisation of mitochondria in young *A. niger* hyphae is not dependent on *secG*, *ageB*, or *geaB* expression.

**Additional file 5.** DNA primers used in this study.

**Additional file 6.** Molecular verification of transformants generated in this study.

## Data Availability

The data sets, strains used and/or analysed during the current study, and sequences are available from the corresponding authors on reasonable request.

## Abbreviations

Arf: ADP ribosylation factors
CM: complete medium
BLAST: basic local alignment search tool
Cas: CRISPR-associated
CRISPR: Clustered Regularly Interspaced Short Palindromic Repeats
.csv: comma-separated values
Dox: doxycycline
Hyg: hygromycin
MN: morphology number
MM: minimal medium
MPD: Morphology of Pelleted and Dispersed growth
sg: synthetic guide
Tet: tetracycline
TCA: tricarboxylic acid
