# Correction to: Functional exploration of co-expression networks identifies a nexus for modulating protein and citric acid titres in *Aspergillus niger* submerged culture

**DOI:** 10.1186/s40694-019-0087-4

**Published:** 2019-12-17

**Authors:** Timothy C. Cairns, Claudia Feurstein, Xiaomei Zheng, Li Hui Zhang, Ping Zheng, Jibin Sun, Vera Meyer

**Affiliations:** 1 Tianjin Institute of Industrial Biotechnology, Chinese Academy of Sciences, Tianjin, 300308 People’s Republic of China; 2 Key Laboratory of Systems Microbial Biotechnology, Chinese Academy of Sciences, Tianjin, 300308 People’s Republic of China; 3 Institute of Biotechnology, Chair of Applied and Molecular Microbiology, Technische Universität Berlin, Straße des 17. Juni 135, 10623 Berlin, Germany; 4 University of Chinese Academy of Sciences, Beijing, 100049 China; 5 College of Biotechnology, Tianjin University of Science & Technology, Tianjin, 300457 China

## Correction to: Fungal Biol Biotechnol (2019) 6:18 10.1186/s40694-019-0081-x

Prior to publication of the original article [1], the authors provided revised images for Figs. 4, 6 and 7 during the proof-correction stage. These were not processed by the typesetter. The corrected Figs. 4, 6 and 7 are given with this erratum.

**Fig. 4:**
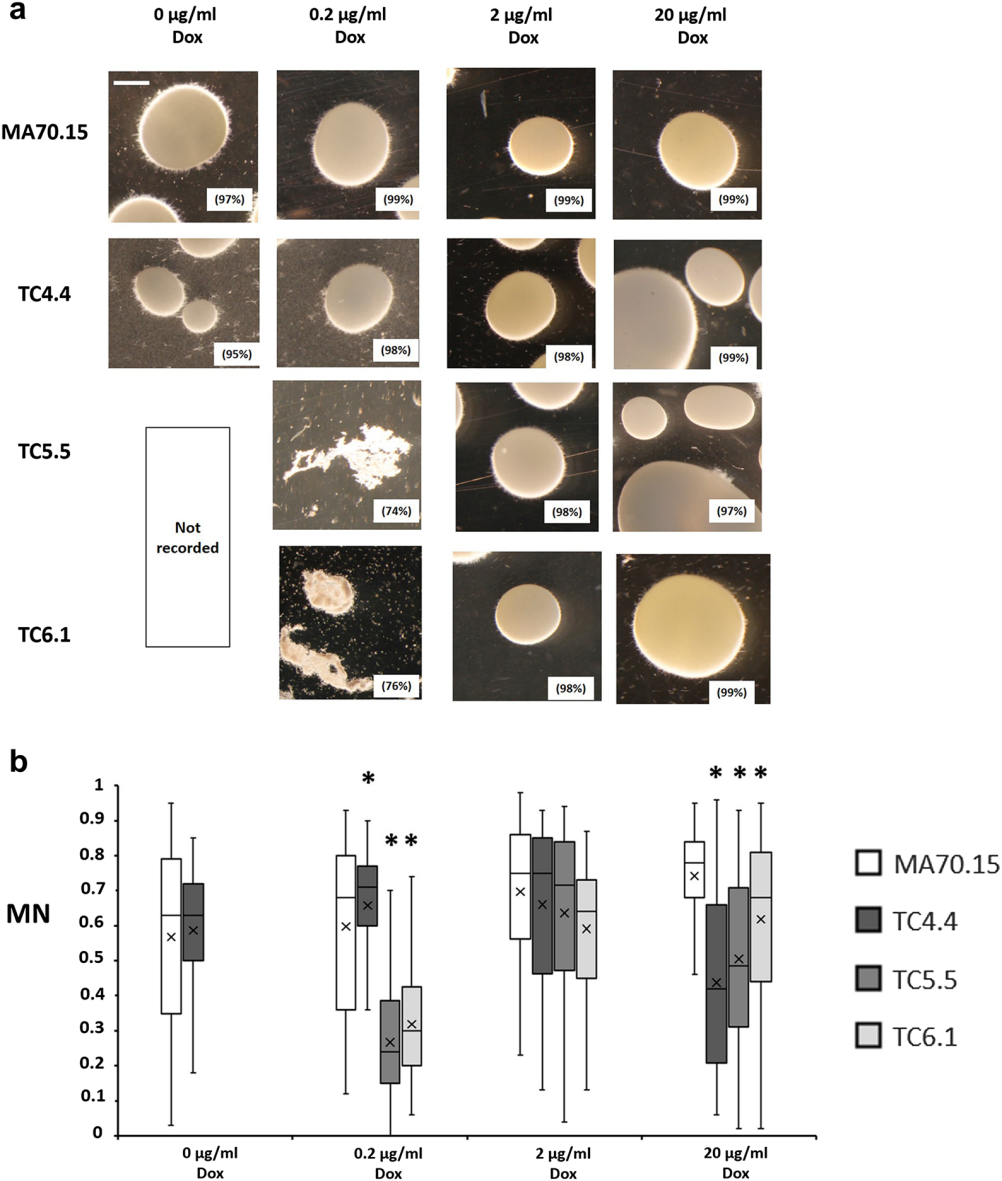
Representative images and quantitative analysis of conditional expression mutant macromorphology during submerged growth in minimal media. To model protein fermentation conditions, 1 × 10^6^ spores/ml of conditional expression mutants and progenitor control (MA70.15) were inoculated in 20 ml MM with 5% glucose as carbon source and supplemented with various concentrations of Dox. Cultures were grown at 220 RPM, 30 °C, for 72 h. **a** Representative images are depicted for triplicated experiments each consisting of duplicate replicates. Pelleted morphologies (any fungal structure > 500 µm^2^ area) are reported as a function of the total fungal area measured during image analysis, and are indicated as a percentage in parenthesis. Scale bar in the top left panel is 1 mm. **b** Shake flask cultures were quantitatively analysed using the MPD image analysis pipeline [37]. Reported are box whisker plots for pellet morphology number (MN). Crosses depict average values. Pairwise Student‘s *t*-tests were conducted between conditional expression mutant relative to the MA70.15 control at respective Dox concentrations. *p* values are indicated as (< 0.05, *)

**Fig. 6:**
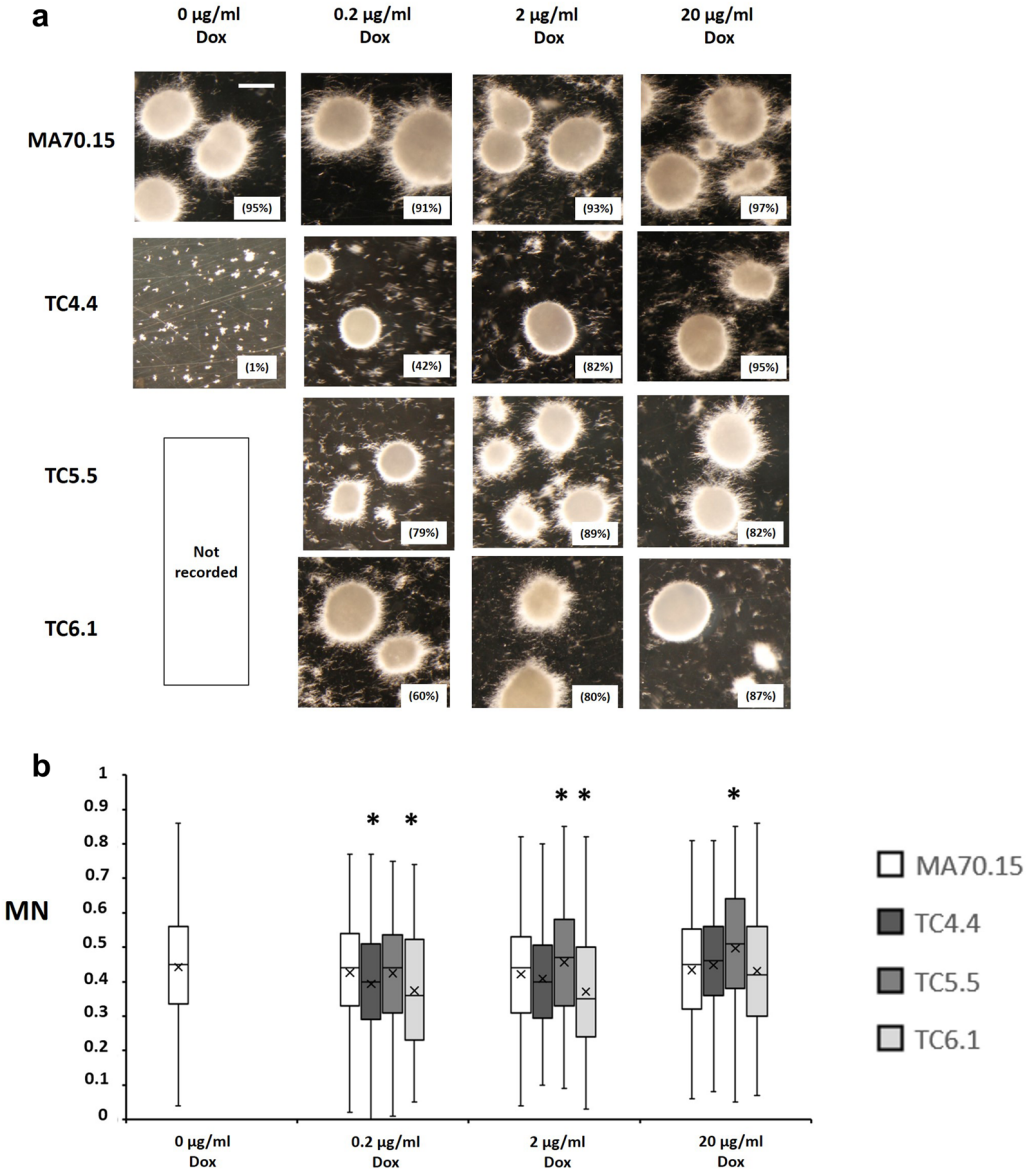
Representative images and quantitative analysis of conditional expression mutant macromorphology during submerged growth in citric acid production media. 1 × 10^5^ spores/ml of each isolate were inoculated in organic acid production medium CitACM with 10% sucrose as carbon source. Cultures were grown at 220 RPM, 34 °C, for 96 h (see “Methods” section for full conditions). Representative images are depicted for triplicated experiments each consisting of duplicate replicates (**a**). Pelleted morphologies (any fungal structure > 500 µm^2^ area) are reported as a function of the total fungal area measured during image analysis, and are indicated as a percentage in parenthesis. Scale bar in the top left panel is 1 mm. Shake flask cultures were quantitatively analysed (**b**) using the MPD image analysis pipeline as described in Fig. 4b. Note that pelleted morphologies were almost entirely absent in the *secG* mutant TC4.4 0 µg/ml Dox during growth in organic acid production medium

**Fig. 7:**
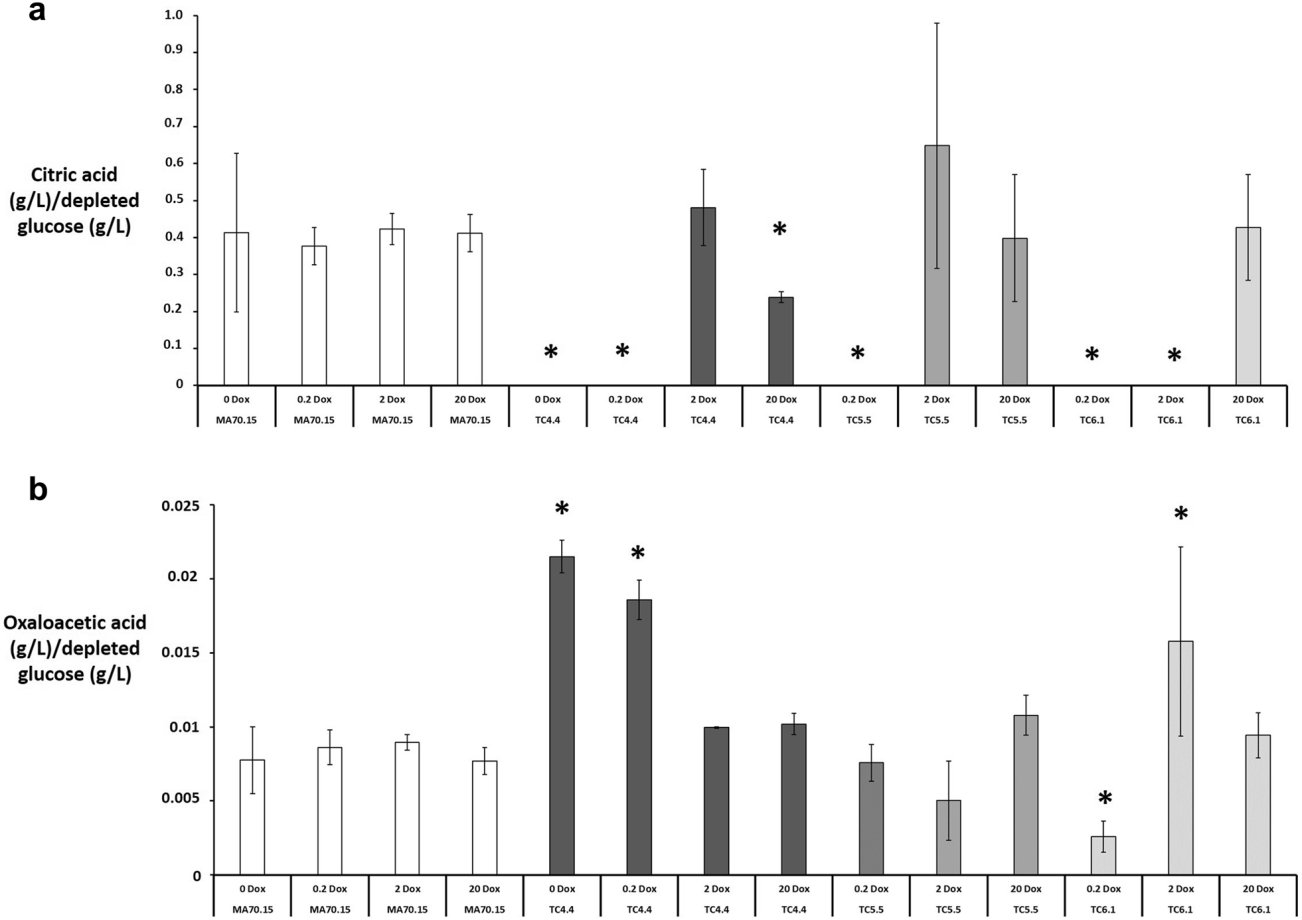
*A. niger* genes *secG*, *ageB*, and *geaB* have critical impacts on organic acid titres during submerged culture. From organic acid production medium in shake flask cultures, secreted citric acid and oxaloacetate were quantified using HPLC, and normalised to depleted glucose. Pairwise Student‘s *t*-tests were conducted between conditional expression mutant relative to the MA70.15 control at respective Dox concentrations (μg/ml). *p* values are indicated as (< 0.05, *). Note that mutants TC4.5 and TC5.6 performed comparably to their isogenic comparator, and are omitted from this figure for clarity

The publishers apologise for this error. The original article has been updated.
