# Supplementary material for: Functional exploration of co-expression networks identifies a nexus for modulating protein and citric acid titres in Aspergillus niger submerged culture
Source: Fungal Biol Biotechnol. 2019 Nov 9;6:18. doi: 10.1186/s40694-019-0081-x (PMC6842248; doi:10.1186/s40694-019-0081-x)
Supplement: Supplementary file 3 — Additional file 3. Conservation between yeast GEF and GAP domains with predicted A. niger AgeB, SecG, and GeaB proteins. [file 40694_2019_81_MOESM3_ESM.docx]

**Functional exploration of co-expression networks identifies a nexus for modulating protein and citric acid titres in *Aspergillus niger* submerged culture**

**Timothy C. Cairns^1,2^, Claudia Feurstein^1,2,3^, Xiaomei Zheng^1,2,4^, Li Hui Zhang^1,2,5^, Ping Zheng^1,2,4^, Jibin Sun^1,2,4^, and Vera Meyer^1,2,3,4^**

^1^ Tianjin Institute of Industrial Biotechnology, Chinese Academy of Sciences, Tianjin, 300308, People’s Republic of China

^2^ Key Laboratory of Systems Microbial Biotechnology, Chinese Academy of Sciences, Tianjin 300308, People’s Republic of China

^3^Technische Universität Berlin, Institute of Biotechnology, Chair of Applied and Molecular Microbiology,

Straße des 17. Juni 135, 10623 Berlin, Germany

^4^ University of Chinese Academy of Sciences, Beijing, 100049 China

^5^ College of Biotechnology, Tianjin University of Science & Technology, Tianjin, 300457 China

Timothy C. Cairns: t.cairns@tu-berlin.de

Claudia Feurstein: c.feurstein@tu-berlin.de

Li Hui Zhang: zhanglh@tib.cas.cn

Xiaomei Zheng: zheng_xm@tib.cas.cn

Jibin Sun: sun_jb@tib.cas.cn

Ping Zheng: zheng_p@tib.cas.cn

Vera Meyer: [vera.meyer@tu-berlin.de](mailto:vera.meyer@tu-berlin.de), ORCID 0000-0002-2298-2258

Contact details for corresponding authors:

Vera Meyer, Tel.: +49 30 314 72750, Fax: +49 30 314 72922, E-mail: [vera.meyer@tu-berlin.de](mailto:vera.meyer@tu-berlin.de)

Sun, Tel.: +86-8486 1949, Fax: +86-8486 1943, E-mail: [sun_jb@tib.cas.cn](mailto:sun_jb@tib.cas.cn)


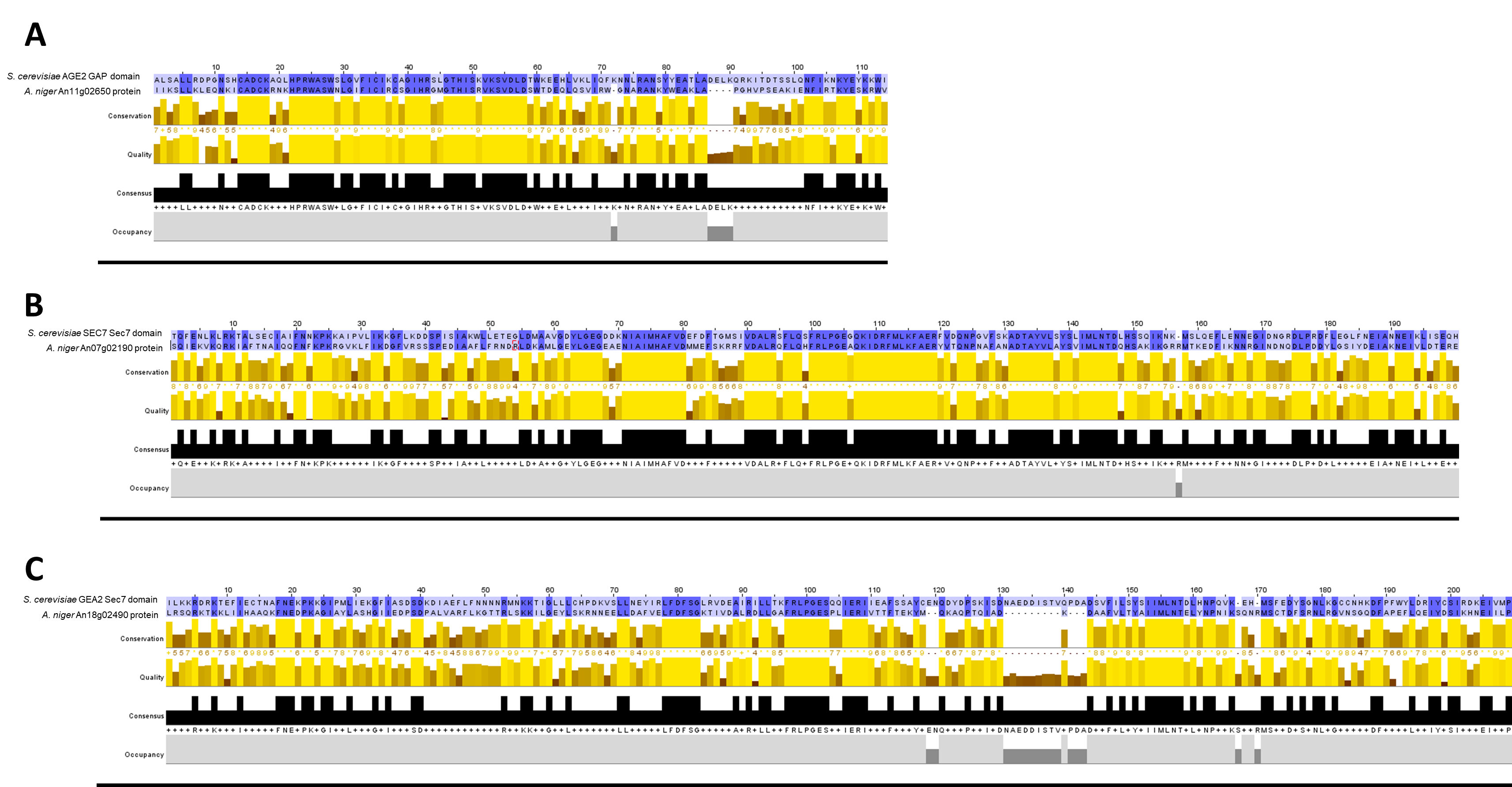


**Supplementary File 3: Conservation between yeast GEF and GAP domains with predicted *A. niger* Age1, Sec7, and Gea2 proteins.** *S. cerevisiae* domains were retrieved from the Saccharomyces genome database, whereas *A. niger* ORF sequences were downloaded from the Ensembl database. Pairwise alignments were performed with JalView Version 2 using default parameters. Total sequence conservation for *A. niger* Age1 (A), Sec7 (B), and Gea2 (C) and respective yeast domains was 51.8%, 55.5%, and 38.2%, respectively
