# Supplementary material for: Functional exploration of co-expression networks identifies a nexus for modulating protein and citric acid titres in Aspergillus niger submerged culture
Source: Fungal Biol Biotechnol. 2019 Nov 9;6:18. doi: 10.1186/s40694-019-0081-x (PMC6842248; doi:10.1186/s40694-019-0081-x)
Supplement: Supplementary file 4 — Additional file 4. Localisation of mitochondria in young A. niger hyphae is not dependent on secG, ageB, or geaB expression. [file 40694_2019_81_MOESM4_ESM.docx]

**Functional exploration of co-expression networks identifies a nexus for modulating protein and citric acid titres in *Aspergillus niger* submerged culture**

**Timothy C. Cairns^1,2^, Claudia Feurstein^1,2,3^, Xiaomei Zheng^1,2,4^, Li Hui Zhang^1,2,5^, Ping Zheng^1,2,4^, Jibin Sun^1,2,4^, and Vera Meyer^1,2,3,4^**

^1^ Tianjin Institute of Industrial Biotechnology, Chinese Academy of Sciences, Tianjin, 300308, People’s Republic of China

^2^ Key Laboratory of Systems Microbial Biotechnology, Chinese Academy of Sciences, Tianjin 300308, People’s Republic of China

^3^Technische Universität Berlin, Institute of Biotechnology, Chair of Applied and Molecular Microbiology,

Straße des 17. Juni 135, 10623 Berlin, Germany

^4^ University of Chinese Academy of Sciences, Beijing, 100049 China

^5^ College of Biotechnology, Tianjin University of Science & Technology, Tianjin, 300457 China

Timothy C. Cairns: t.cairns@tu-berlin.de

Claudia Feurstein: c.feurstein@tu-berlin.de

Li Hui Zhang: zhanglh@tib.cas.cn

Xiaomei Zheng: zheng_xm@tib.cas.cn

Jibin Sun: sun_jb@tib.cas.cn

Ping Zheng: zheng_p@tib.cas.cn

Vera Meyer: [vera.meyer@tu-berlin.de](mailto:vera.meyer@tu-berlin.de), ORCID 0000-0002-2298-2258

Contact details for corresponding authors:

Vera Meyer, Tel.: +49 30 314 72750, Fax: +49 30 314 72922, E-mail: [vera.meyer@tu-berlin.de](mailto:vera.meyer@tu-berlin.de)

Sun, Tel.: +86-8486 1949, Fax: +86-8486 1943, E-mail: [sun_jb@tib.cas.cn](mailto:sun_jb@tib.cas.cn)


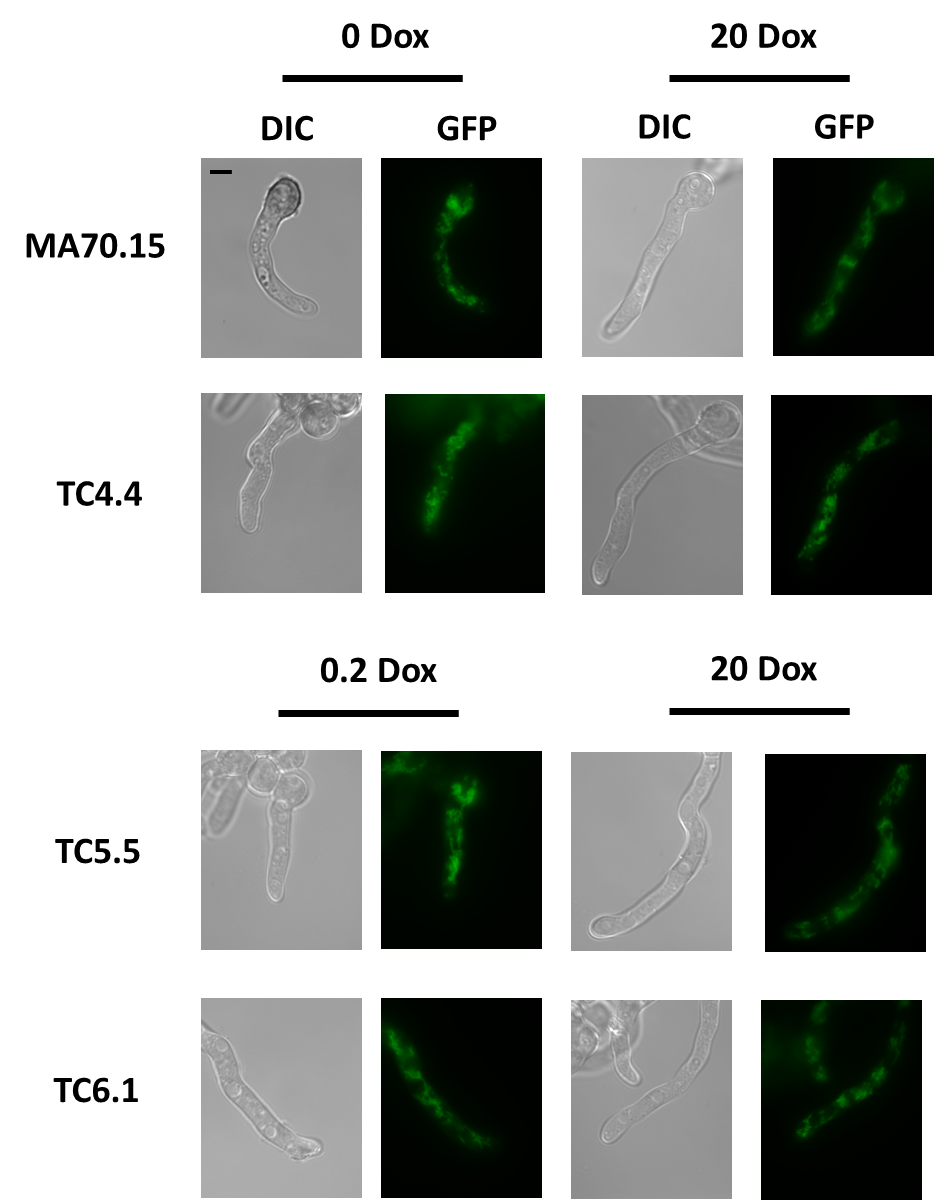


**Additional File 4: Localisation of mitochondria in young *A. niger* hyphae is not dependent on *secG*, *ageB*, or *geaB* expression.** Isolates were inoculated in CitACM media supplemented with the indicated Dox concentration and grown for 8 hours at 34°C with 220 RPM. Cells were stained with MitoTracker Green FM (Thermo Fisher, Germany) and microscopy conducted using an inverted TCS SP8 fluorescent microscope (Leica, Germany). This assay did not detect any differences in mitochondrial localisation between conditional expression mutants and control, or between 0/20 µg/ml Dox and 0.2/20µg/ml Dox concentrations. Scale bar in top left panel is 5 µm.
