# Supplementary material for: Functional exploration of co-expression networks identifies a nexus for modulating protein and citric acid titres in Aspergillus niger submerged culture
Source: Fungal Biol Biotechnol. 2019 Nov 9;6:18. doi: 10.1186/s40694-019-0081-x (PMC6842248; doi:10.1186/s40694-019-0081-x)
Supplement: Supplementary file 6 — Additional file 6. Molecular verification of transformants generated in this study. [file 40694_2019_81_MOESM6_ESM.docx]

**Functional exploration of co-expression networks identifies a nexus for modulating protein and citric acid titres in *Aspergillus niger* submerged culture**

**Timothy C. Cairns^1,2^, Claudia Feurstein^1,2,3^, Xiaomei Zheng^1,2,4^, Li Hui Zhang^1,2,5^, Ping Zheng^1,2,4^, Jibin Sun^1,2,4^, and Vera Meyer^1,2,3,4^**

^1^ Tianjin Institute of Industrial Biotechnology, Chinese Academy of Sciences, Tianjin, 300308, People’s Republic of China

^2^ Key Laboratory of Systems Microbial Biotechnology, Chinese Academy of Sciences, Tianjin 300308, People’s Republic of China

^3^Technische Universität Berlin, Institute of Biotechnology, Chair of Applied and Molecular Microbiology,

Straße des 17. Juni 135, 10623 Berlin, Germany

^4^ University of Chinese Academy of Sciences, Beijing, 100049 China

^5^ College of Biotechnology, Tianjin University of Science & Technology, Tianjin, 300457 China

Timothy C. Cairns: t.cairns@tu-berlin.de

Claudia Feurstein: c.feurstein@tu-berlin.de

Li Hui Zhang: zhanglh@tib.cas.cn

Xiaomei Zheng: zheng_xm@tib.cas.cn

Jibin Sun: sun_jb@tib.cas.cn

Ping Zheng: zheng_p@tib.cas.cn

Vera Meyer: [vera.meyer@tu-berlin.de](mailto:vera.meyer@tu-berlin.de), ORCID 0000-0002-2298-2258

Contact details for corresponding authors:

Vera Meyer, Tel.: +49 30 314 72750, Fax: +49 30 314 72922, E-mail: [vera.meyer@tu-berlin.de](mailto:vera.meyer@tu-berlin.de)

Sun, Tel.: +86-8486 1949, Fax: +86-8486 1943, E-mail: [sun_jb@tib.cas.cn](mailto:sun_jb@tib.cas.cn)


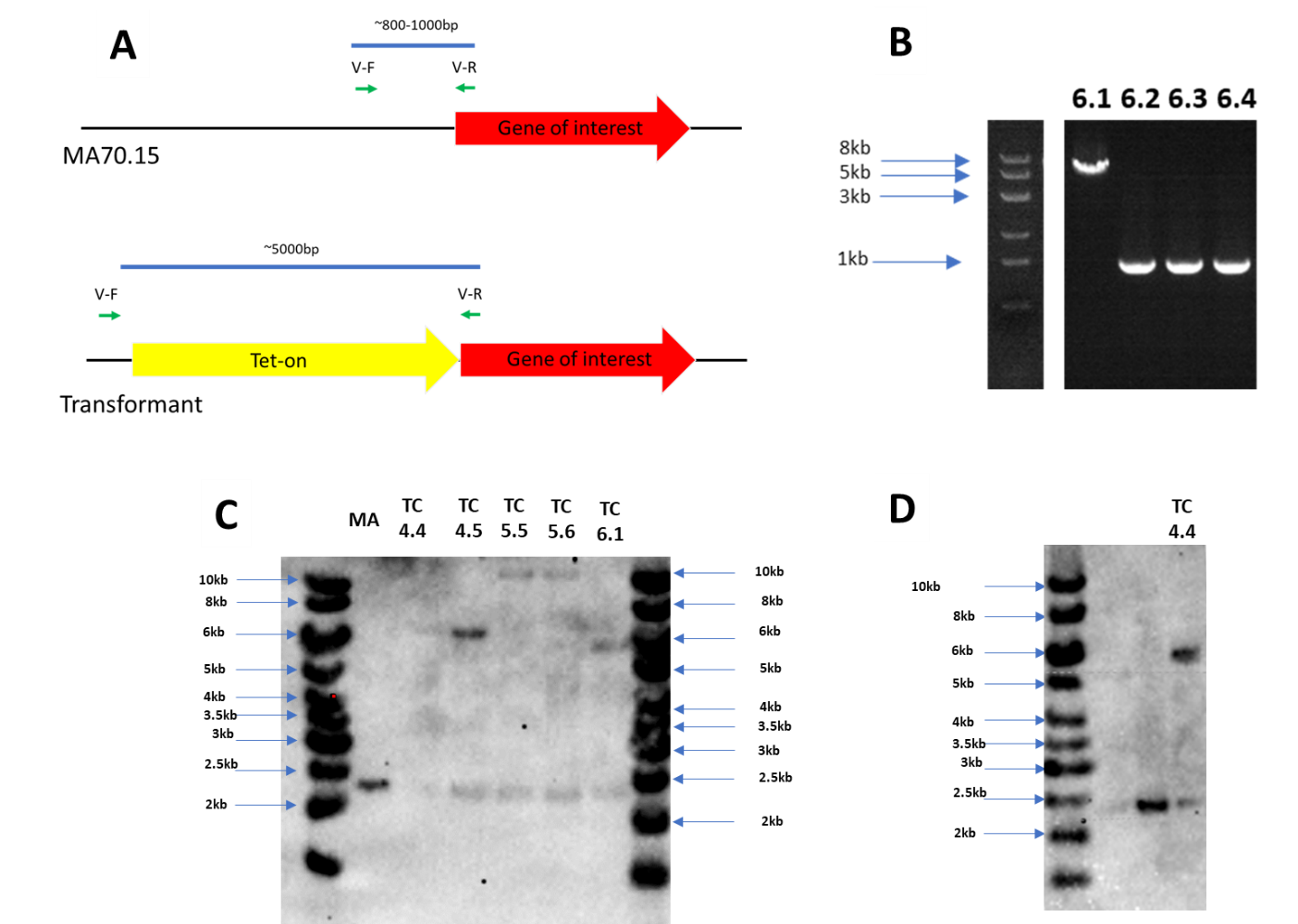


**Supplementary File S6: Molecular verification of transformants generated in this study.** Schematic representation (A) of PCR confirmation strategy using verification primers Gene-V-F and Gene-V-R (Supplementary File S5). In this approach, PCR amplicons are generated with a size of 0.8-1 kb (MA70.15 genomic DNA) or ~5kb (transformant genomic DNA) due to gene editing of the Tet-on cassette upstream of the target gene. Approximate primer locations are given with green arrows. Exemplar PCR verification of transformant TC6.1 and unsuccessful transformants (TC6.2, TC6.3, TC6.4) are given (B). Transformants which passed PCR verification were analysed by Southern blot (C and D). A 1 kb portion of the *fraA* promoter locus (An16g04690) was labelled by PCR amplification using primers listed in Supplementary File 5. Genomic DNA from progenitor strain MA70.15 (MA) or conditional expression mutants was extracted and digested with PvuII and HindIII. During Southern blot hybridisation, the native An16g04690 locus results in a 2.3 kb fragment which served as a positive control for DNA integrity. The addition of the *fraA* promoter present in the Tet-on cassette results in an addition band for TC4.4/TC4.5 (6.6 kb), TC5.5/TC5.6 (10.8 kb) and TC6.1 (5.9 kb). Note that due to poor DNA integrity for isolate TC4.4 in blot (C), a technical replicate was conducted (D).
